# Recent Advances of COVID-19 Modeling Based on Regenerative Medicine

**DOI:** 10.3389/fcell.2021.683619

**Published:** 2021-10-25

**Authors:** Bagher Larijani, Najmeh Foroughi-Heravani, Mina Abedi, Akram Tayanloo-Beik, Mostafa Rezaei-Tavirani, Hossein Adibi, Babak Arjmand

**Affiliations:** ^1^Endocrinology and Metabolism Research Center, Endocrinology and Metabolism Clinical Sciences Institute, Tehran University of Medical sciences, Tehran, Iran; ^2^Cell Therapy and Regenerative Medicine Research Center, Endocrinology and Metabolism Molecular-Cellular Sciences Institute, Tehran University of Medical Sciences, Tehran, Iran; ^3^Metabolomics and Genomics Research Center, Endocrinology and Metabolism Molecular-Cellular Sciences Institute, Tehran University of Medical Sciences, Tehran, Iran; ^4^Proteomics Research Center, Shahid Beheshti University of Medical Sciences, Tehran, Iran; ^5^Diabetes Research Center, Endocrinology and Metabolism Clinical Sciences Institute, Tehran University of Medical Sciences, Tehran, Iran

**Keywords:** COVID-19, SARS-CoV-2, model, stem cell, induced pluripotent stem cells, regenerative medicine, organoid

## Abstract

Severe Acute Respiratory Syndrome Coronavirus-2 (SARS-CoV-2) has caused a pandemic since December 2019 that originated in Wuhan, China. Soon after that, the world health organization declared Coronavirus disease-2019 a global health concern. SARS-CoV-2 is responsible for a lethal respiratory infection as well as the involvement of other organs due to its large tropism spectrum such as neurologic, cardiovascular, endocrine, gastrointestinal, and renal systems. Since the behavior of the virus is not fully understood, a new manifestation of the infection is revealed every day. In order to be able to design more efficient drugs and vaccines to treat the infection, finding out the exact mechanism of pathogenicity would be necessary. Although there have been some big steps toward understanding the relevant process, there are still some deficiencies in this field. Accordingly, regenerative medicine (RM), can offer promising opportunities in discovering the exact mechanisms and specific treatments. For instance, since it is not always possible to catch the pathophysiology mechanisms in human beings, several modeling methods have been introduced in this field that can be studied in three main groups: stem cell-based models, organoids, and animal models. Regarding stem cell-based models, induced pluripotent stem cells are the major study subjects, which are generated by reprogramming the somatic stem cells and then directing them into different adult cell populations to study their behavior toward the infection. In organoid models, different cell lines can be guided to produce a 3D structure including liver, heart, and brain-like platforms. Among animal models, mice are the most common species in this field. However, in order for mice models to be permissive to the virus, angiotensin-converting enzyme 2 receptors, the main receptor involved in the pathogenicity of the virus, should be introduced to the host cells through different methods. Here, the current known mechanism of SARS-CoV-2 infection, different suggested models, the specific response toward different manipulation as well as challenges and shortcomings in each case have been reviewed. Finally, we have tried to provide a quick summary of the present available RM-based models for SARS-CoV-2 infection, as an essential part of developing drugs, for future therapeutic goals.

## Introduction

Emerging from Wuhan, China, severe acute respiratory syndrome Coronavirus-2(SARS-CoV-2) has introduced a new pandemic to the world. Coronavirus disease-2019(COVID-19) is the new deadly viral infection in the family of human coronaviruses including SARS and Middle East Respiratory Syndrome (MERS). It is more contagious than the former ones and has caused considerable mortality and morbidity. Due to the lack of an effective treatment, the number of patients is rising constantly (Akhmerov and Marbán, 2020; Basiri et al., 2020; Becker, 2020; Golchin et al., 2020; Yang et al., 2020).

Scientists’ knowledge of this infection is rapidly growing, for instance, the function of the angiotensin-converting enzyme2 (ACE2) receptor in SARS-CoV-2 tropism and mechanism of infection is partially understood (Harmer et al., 2002; Becker, 2020; Yu et al., 2020). The ACE2 receptor is expressed on a large proportion of human cells such as lung parenchyma, the heart, kidney, and gastrointestinal tract. It is believed that the ACE2 receptor plays a significant role in the presentation of many symptoms: acute respiratory distress syndrome (ARDS), diarrhea, etc. (Yiangou et al., 2020). Although many biotechnology companies have developed different vaccines and millions of people have been vaccinated to date, their effectiveness is still under question and longer follow-up is needed. Among various attempts and intensive research on possible strategies, constructing regenerative medicine (RM) based platforms has been investigated for a novel therapeutic approach that can provide the information needed for understanding the virus behavior and its pathogenesis (Ramezankhani et al., 2020; Yang et al., 2020). RM is about repairing, regenerating, and restoring the missing function of organs or tissues, thus it can be beneficial for studying the interactions between the virus and host cells and therefore, the pathophysiology, which can lead to the development of new drugs and vaccines (Atala et al., 2020; Basiri et al., 2020; Yang et al., 2020). As an example of therapeutic attempts, cell therapies, especially mesenchymal stem cell (MSC) therapy, have undergone investigations. MSCs have promising features like immunomodulatory effects, which are helpful in treating SARS-CoV-2 infection (Basiri et al., 2020; Golchin et al., 2020). At the time of writing this review, around 80 clinical trials are registered in *www.clinicaltrials.gov* that have utilized cell- based strategies including stem cells (mostly MSCs) and their derivatives (e.g., exosomes), memory T cells, and natural killer (NK) cells for treating COVID-19 and its related organ injuries. However, there are limited data on MSC therapy in pre-clinical studies, especially on models of lung injury of COVID-19 (Khoury et al., 2020; Ramezankhani et al., 2020; Ferreras et al., 2021; Park et al., 2021; Pérez-Martínez et al., 2021). Disease modeling (*in vitro, in vivo*, or both) is considered to be one of the major components of RM. Indeed, to elucidate the infection mechanisms and its manifestation in the human body, proper and reliable models are of urgent need. Accordingly, many animal models, different types of stem cells, and the cell-based cultures and organoids [a 3 dimensional (3D) structure in extracellular matrix (ECM)] have been used frequently to model diseases including neurological, cardiac, and metabolic disorders (Sterneckert et al., 2014; Doss and Sachinidis, 2019; Atala et al., 2020; Basiri et al., 2020; Sun et al., 2020; Payab et al., 2021). To model COVID-19, some of the most common animal species are mice, rats, and hamsters. For instance, mice have been genetically engineered to express the human ACE2 gene. In addition, there have been attempts toward using ACE2 expressing stem cells in RM platforms (Atala et al., 2020; Yang et al., 2020). Moreover, since SARS-CoV-2 affects multiple systems and organs, RM has played a role in analyzing the infection mechanism and host response by generating organoids of the lung, kidney, cardio, intestine which replicate the critical features of organs and model the disease *in vitro* (Atala et al., 2020; Basiri et al., 2020; Sun et al., 2020). In this review, a brief report of the known pathophysiology of SARS-CoV-2 infection in different organs is presented. Then, an extended review of recent advances toward designing models of COVID-19 is provided to help researchers find the best and most appropriate model. This article has tried to present the available data on modeling strategies of this new infection and their pros and cons for designing future effective therapeutic strategies.

## Mechanism of SARS-CoV-2 Infection

Severe Acute Respiratory Syndrome Coronavirus-2 (SARS-CoV-2) is a highly transmissible virus that demonstrates a broad spectrum of tissue tropism, a quality that justifies its level of contagiousness (Harrison et al., 2020; Khoury et al., 2020). Due to its multi-organ tropism, a spectrum of pathologic symptoms from neurological, cardiac, pancreatic, digestive, and renal to the most common one, respiratory signs can be presented (Alsaad et al., 2020; Puelles et al., 2020; Qin and Zhao, 2020; Simoneau and Ott, 2020; Wiersinga et al., 2020; Yang et al., 2020). The multi-organ tropism is due to the ACE2 receptor expression on different cells around the body. In the following part, a brief description of the chain of events when SARS-CoV-2 attacks different organs of the body is presented. Knowing this mechanism helps us understand the foundation of each model designing better.

### Lung

In respiratory tract involvement, SARS-CoV-2 causes symptoms from viral pneumonia to ARDS of the upper and lower respiratory tracts (Khoury et al., 2020; Simoneau and Ott, 2020; Youk et al., 2020). ARDS diagnosis is confirmed based on Berlin 2012 criteria: (1) presence of a clinical insult as well as new or worsening respiratory symptoms. (2) bilateral opacities on the imaging that are not fully described as effusion, collapse, or nodule. (3) respiratory failure due to edema that is not due to cardiac failure or volume overload and (4) impaired oxygenation that is described in three levels of mild, moderate, and severe each with its specific cut-offs (Gibson et al., 2020). Since the beginning of the pandemic in 2019, there have been some huge steps toward discovering the mechanism of action and virulence of the virus in order to improve the efficacy of vaccines and drug candidates (Ramezankhani et al., 2020; Zheng et al., 2021). SARS-CoV-2 is composed of four main structural proteins including Spike (S), Membrane (M), Envelope (E), and Nucleocapsid (N). In the mechanism of virus infection, the very first step of pathogenicity is the entrance to the parenchymal lung cells by the interaction between ACE2 receptors and virus surface S protein (Harrison et al., 2020; Li et al., 2020; Ramezankhani et al., 2020; Shang et al., 2020; Simoneau and Ott, 2020; Yang et al., 2020). After that, viral S protein undergoes lysis by transmembrane serine protease 2 (TMPRSS2) of the host cells. This gives the virus genome an opportunity to be translated to viral polymerase and proteases by the host ribosomes (Li et al., 2020; Simoneau and Ott, 2020). The products of this process are viral RNA and structural proteins (Fehr and Perlman, 2015; Li et al., 2020; Simoneau and Ott, 2020). The range of SARS-CoV-2 effects on the respiratory system varies from pneumonia to ARDS. The clinical definition was provided in the previous part. In SARS-CoV-2 infection, the pathological features of ARDS are associated with diffuse alveolar changes such as hyaline membrane formation, interstitial thickening, edema, and fibroblasts proliferation. The associated mechanisms are discussed in the following (Gibson et al., 2020). One of them is the direct cytopathic effect (Ramezankhani et al., 2020). Studies have shown that SARS-CoV-2 causes apoptosis and necrosis in ACE2 positive cells (airway epithelial cells in this case) via cytopathic effect. In addition, cilia movement cessation is another pathologic effect in the airways. Direct virus entrance in some other ACE2 positive cells is one of the possible suggested mechanisms of cell damage (Ramezankhani et al., 2020; Wang et al., 2020). Considering the immunologic effects, the host retinoic acid-inducible gene-I-like (RIG-I) or Toll-like receptors recognize viral pathogen-associated molecular patterns (PAMPs) (Simoneau and Ott, 2020). In the cellular immune system, antigen presenting cells (APC) activate CD4 + cells as well as CD8 + T cells, which destroy infected cells directly. Furthermore, a part of the pro-inflammatory cytokine production (such as interleukin (IL)-1, IL-6, IL-12, interferon(IFN)-γ, and Tumor Necrosis Factor(TNF)-a) is induced by CD4 + T cells, that leads to recruitment of innate immune cells (Costela-Ruiz et al., 2020; Ramezankhani et al., 2020). The uncontrolled production of inflammatory cytokines can also cause cytokine storm, which then leads to ARDS (Molinaro et al., 2020; Ramezankhani et al., 2020). The cytokine storm increases vascular permeability leading to edema as one of the items in ARDS criteria due to damaging the endothelial cell junctions (tight junction protein and the zonula occludens) as well as letting proteins, neutrophils, erythrocytes, and platelets pass into the interstitial tissue. This process is compatible with opacities seen in imaging. Then with the progression of alveolar accumulation with exudate, ventilation to perfusion mismatch is exaggerated and deteriorates impaired oxygenation that results in the progression toward ARDS (Molinaro et al., 2020; Ramezankhani et al., 2020; Suri et al., 2021). Another reason for the hyperinflammatory state is macrophage hyperactivation and neutrophil infiltration. macrophage hyperactivation causes macrophage activation syndrome (MAS) and the latter results in necroinflammation (Cao, 2020; Otsuka and Seino, 2020; Ramezankhani et al., 2020). Interferons (mainly IFN-1) are another group of antiviral factors produced because of antiviral immune response (Boudewijns et al., 2020; Busnadiego et al., 2020; Lei et al., 2020; Simoneau and Ott, 2020). Memory T cells mostly show an immune response to SARS-CoV-2 structural proteins, especially S proteins. Accordingly, structural proteins could be considered as vaccine candidates (Ng et al., 2016; Ramezankhani et al., 2020). SARS-CoV-2 interferes with immune system antiviral actions by using different methods such as causing cell apoptosis and cytopathic effects (Simoneau and Ott, 2020). In addition, SARS-CoV-2 contains enzymes that modify viral and host cell RNAs. This mechanism makes the virus able to escape antiviral receptor detection. Different mechanisms have been proved to be associated with immune system evasion by SASR-CoV-2. SARS-CoV-2 possesses several proteins such as the NSP family, ORF proteins, M, N that are important in evading the immune system through several ways like cleaving host mRNA, inhibiting inflammatory cytokines production, sequestering viral RNA, inducing apoptosis or direct cytopathic effects, and inhibiting host proteins translation. Discussing these mechanisms in detail is beyond the scope of this article (Kasuga et al., 2021). Different factors have been known to be associated with poor prognosis of ARDS in COVID-19 patients, lymphopenia and cytokine storm are the two most important factors (Pourbagheri-Sigaroodi et al., 2020). Cell reduction in lymphopenia happens in T CD4 + and 8 + as well as B cells. The expression of CD38 and human leukocyte adhesion (HLA)-DR in T cells as the hallmark of T cell activation during viral pneumonia, was significantly higher in COVID-19 patients (Hue et al., 2020). PD-1 expression demonstrates immune dysfunction during sepsis. A study showed that during the pathogenesis of the infection, CD8 + T cells express lower levels of PD-1 in comparison to non-COVID ARDS patients (Hue et al., 2020). This finding supports the previous claim that immune system hyper activation is one of the associated mechanisms in ARDS development and probably forecasts its severity during COVID-19 infection (Hue et al., 2020). Higher levels of IL-10, CXCL10/IP-10, granulocyte-macrophage colony-stimulating factor (GM-CSF), and CX3CL1 as well as more severe viral shedding in the examination of nasopharyngeal swabs were also reported in COVID-ARDS patients (Hue et al., 2020). It highlights that immune dysregulation and more severe viral shedding are other negative prognostic factors of COVID-ADRS (Hue et al., 2020). Systemic inflammatory response, as well as multi-organ failure, can affect the susceptibility of developing ARDS. Comorbidities like old age, hypertension, diabetes mellitus, elevated BMI, cardiac and chronic lung diseases are associated with progression to ARDS in COVID-19 infection (Suri et al., 2021). On the other hand, some studies have reported a more severe phenotype of the disease in immunocompromised patients (Belsky et al., 2021). Therefore, we can conclude that hyper inflammatory response of a competent immune system and the cytopathic effect of the virus in the absence of an effective immune system are two possible underlying factors of developing ARDS. However, this deduction is not complete due to the lack of sufficient knowledge in the case of COVID-19 pathogenicity, which reflects the need for designing effective models in order to facilitate studying viral mechanisms of action in more detail (Remy et al., 2020).

### Kidney

Renal involvement in COVID-19 infection is manifested most commonly as Acute Kidney Injury (AKI) which makes the patient prone to other complications (Robbins-Juarez et al., 2020; Arikan et al., 2021). Several pathophysiologic mechanisms have been suggested to be the cause of COVID-19 renal manifestations, which are discussed briefly in the following point. The first one is systemic hemodynamic instability (Legrand et al., 2021). In this state, due to decreased cardiac output or renal venous congestion, renal perfusion is disrupted. The underlying reasons for decreased cardiac output are mentioned under the cardiovascular system subtitle. On the other hand, venous dilation increases renal interstitial and tubular pressure that lead to a hypoxic situation as well as compromising the glomerular filtration rate (Legrand et al., 2021). Another underlying reason is cytokine storm, a life-threatening situation during which the immune system is highly activated and inflammatory cytokines are extensively released, causing organ failure. In addition to systemic inflammation, cytokine release can happen locally in renal tissue. During COVID-19 infection, renal cells start to release inflammatory cytokines such as TNF and FAS that cause renal dysfunction by direct cell injury (Legrand et al., 2021). The virus can also induce cell damage through the cytopathic effect that invades renal cells directly. On the other hand, SARS-CoV-2 inhibits type I IFN production which leads to increased viral replication as well as immune dysregulation. Out-of-control complement release is another underlying reason for hyper-inflammatory state during SARS-CoV-2 infection that induces tissue injury. Adaptive immunity dysfunction such as T cell, plasmacytoid dendritic cell, eosinophil, and natural killer cell depletion has also been reported during the course of infection. The last mechanism is endothelial damage and micro-thrombi formation, the pathophysiology of which is discussed under the “cardiovascular system subtitle” (Legrand et al., 2021).

### Eye

According to studies, COVID-19 infection does not cause specific retinal involvement, however, conjunctivitis has been reported in some cases with positive PCR (Pirraglia et al., 2020; Seah and Agrawal, 2020). On the other hand, patients with eye comorbidities are at risk of advanced or uncontrolled forms of the disease because of disrupted ophthalmic care delivery during the pandemic (Pujari et al., 2021). However, several ophthalmic symptoms have been reported in animal studies such as retinitis, conjunctivitis, anterior uveitis, chorditis, retinal detachment and, optic neuritis (Seah and Agrawal, 2020). The underlying vacuities during the COVID-19 infection can cause these symptoms (Seah and Agrawal, 2020). Tissue inflammation is also directly induced by viral replication in the retina that causes immune cells to infiltrate and pro-inflammatory cytokines to be released (Seah and Agrawal, 2020). An autoimmune nature of the infection is also suggested in the studies due to the autoantibodies that are produced during the infection against retinal cells that lead to degradation of photoreceptors, ganglion cells, and neuroretina (Seah and Agrawal, 2020). ACE2 protein, which is necessary for the virus entrance, is expressed in the aqueous humor but more studies are needed in order to explore its expression in other structures such as conjunctiva or cornea (Seah and Agrawal, 2020).

### Gut

Patients with COVID-19 can show several gastrointestinal symptoms such as diarrhea, nausea, vomiting, gastrointestinal bleeding, and abdominal pain (Zhang J. et al., 2020). ACE2 receptor, as well as TMPRSS2, are highly expressed in the gastrointestinal system, thus viral entrance and replication happen extensively in the gut after the respiratory system. Tissue inflammation underlies these symptoms during infection (Steardo et al., 2020; Zhang J. et al., 2020). High levels of fecal calprotectin and IL-6 as inflammatory factors support this claim (Zhang J. et al., 2020). Hyper-inflammatory syndromes such as hemophagocytic lymphohistiocytosis and cytokine storm also are accused of organ failure in the gastrointestinal system like other body organs (Zhang J. et al., 2020). However, based on studies human defensin-5 protein that plays an important role against SARS-CoV-2 in the gut, can be increased during the inflammation (Zhang J. et al., 2020).

### Liver

Abnormal liver transferase levels can be detected in about 15–43% of COVID-19 patients with more probability in severe cases (Zhang C. et al., 2020). Acute liver injury has also been reported in some studies (Zhang C. et al., 2020). Liver injury can happen during systemic inflammation that is caused by inflammatory cytokine release during the course of infection. High levels of Th17 and 2, IL-2,6,7,10, TNF-a, granulocyte-colony stimulating factor, IFN-inducible protein-10, monocyte chemotactic protein 1and macrophage inflammatory protein 1 alpha in COVID-19 patients support this idea (Li and Fan, 2020). Liver dysfunction can also happen through direct viral invasion in the infection (Zhang C. et al., 2020). In addition, stress- induced liver injury is another pathologic event that can be caused by hypoxia-reoxygenation, activation of oxidative stress mechanisms, intestinal endotoxemia, and activation of the sympathetic nervous and adrenocortical system in COVID-19 patients (Li and Fan, 2020). As cholangiocytes highly express ACE2 receptors, they are suggested to be one of the main cells responsible for liver injury in COVID-19 infection (Zhang C. et al., 2020). However, ACE2 is poorly expressed in the liver, suggesting that there are other entrance paths of the virus to infect the cells (Steardo et al., 2020). Drug toxicity is also mentioned to be another reason for liver dysfunction (Li and Fan, 2020; Zhang C. et al., 2020).

### Brain

Neurological symptoms of SARS-CoV-2 infection are categorized into two different groups, the first one is anosmia and ageusia that are reported in mild cases and the second group of symptoms consists of mental confusion and cognitive impairment in severe cases (Chigr et al., 2020). ACE2 receptors in the CNS are specifically expressed in the brain stem, subfornical organ, paraventricular nucleus, the nucleus of tractus solitaries, and rostral ventrolateral medulla which are responsible for respiratory and cardiovascular systems regulation, therefore a part of these systems dysfunction can be explained by this claim (Chigr et al., 2020; Steardo et al., 2020). There are different entrance paths to the nervous system. Nasal inoculation of SARS-CoV-2 can infect CNS via the olfactory bulb; other paths are bloodstream (blood-brain barrier (BBB) disruption) and vagus nerve that carries the virus from the respiratory system (Chigr et al., 2020; Steardo et al., 2020). Like other organs, tissue inflammation affects the nervous system. Microglia and astrocyte activation support this issue (Steardo et al., 2020). Astrocytes can be attacked by the virus, cells that play an important role in forming BBB, so by astrocyte involvement, the neuro-infection expands. This situation happens when a systemic cytokine storm is triggered by SARS-CoV-2 (Steardo et al., 2020). BBB disruption leads to neuroinflammation that causes neuronal death (Steardo et al., 2020). Hence, the cognitive disorder, behavioral and personality changes that are reported in severe cases can be explained by this mechanism (Steardo et al., 2020). Persistent neuro-inflammation along with hypoxia is accompanied by more severe presentations such as delirium (Steardo et al., 2020).

### Cardiovascular System

As mentioned before, cardiac function can be compromised during SARS-CoV-2 infection leading to decreased cardiac output and in advanced stages, multi-organ dysfunction. Cardiovascular injury can happen through different mechanisms. The first one as was mentioned before is systemic inflammation which involves the cardiovascular system as well as other tissue (Petrovic et al., 2020). Low level of inflammation is the reason for non-specific viral infection symptoms but in severe cases, if the severe inflammatory response syndrome (SIRS) criteria are met, hemodynamic disorders such as shock, disseminated intravascular coagulopathy, and multi-organ failure will lead to cardiac dysfunction (Petrovic et al., 2020). In rare cases, myocardial injury due to hyper-inflammation is reported during COVID-19 infection that compromises cardiac function. Renin–angiotensin system (RAS) activation in the first stages of SIRS to reverse the situation, is responsible for increasing blood pressure through different mechanisms, one of them is vasoconstriction. At first, this condition helps the cardiovascular system to reverse the situation but by the time, hypertension will increase the burden on the cardiovascular system until the heart cannot compensate for the situation (Petrovic et al., 2020). In addition, viral entrance through ACE2 down- regulates its production. This leads to an imbalance between ACE2 and angiotensinogen II levels that is another reason for cardiovascular failure (Petrovic et al., 2020). Hypercoagulable state of the body in COVID-19 as well as plaque instability can exaggerate cardiovascular failure. Hyper-inflammation is one of the triggers of hypercoagulability by disrupting the hematopoietic system (Petrovic et al., 2020). It also plays a negative role in inducing plaque instability. Elevated levels of catecholamines as a result of inflammation may cause plaque rupture that can lead to acute coronary syndrome (Petrovic et al., 2020). Plaque rupture, by exposing its content (foamy macrophage) as well as smooth muscles, induces micro- thrombi formation that can move to other organs and cause organ dysfunction (Petrovic et al., 2020). IL-6, an inflammatory cytokine, can also be released by smooth muscles and worsen the situation.

## Modeling COVID-19

As the pandemic goes on, the necessity to discover the mechanisms of virulence and injury to cells and tissues becomes more and more critical (Sun et al., 2020; Youk et al., 2020). Since it is not always possible to directly investigate the pathophysiology mechanisms in human beings, several modeling methods have been introduced including organoids, different types of stem cells, and animals (Boudewijns et al., 2020; Li et al., 2020; Shpichka et al., 2020; Sun et al., 2020). Several types of 3D-designed organoids derived from body organs that are targets of SARS-CoV-2 organotropism are discussed. Stem cell models can be categorized into two main groups induced pluripotent stem cells (iPSCs) and non- iPSCs. Various types of animal models, each with the quality of being infected by the virus in an innate manner or by being induced through different methods are discussed extendedly under the subtitle. These models can have several functions other than helping the scientists understand different aspects of COVID-19 pathogenesis such as testing the efficacy of drug and vaccine candidates. Based on a recent update of the National Institutes of Health (NIH) COVID-19 treatment guideline, there are some antiviral, immunosuppressant, and antimalarial drugs that are used during the infection. Remdesivir -the only Food and Drug Administration (FDA)-approved COVID-19 drug- and dexamethasone are approved by the NIH guideline (Covid-19 Treatment Guidelines Panel, 2021). The convalescent plasma is suggested for the emergency cases. In addition, ivermectin, nitazoxanide, hydroxychloroquine, chloroquine, azithromycin, lopinavir/ritonavir, and other HIV Protease inhibitors are some other drugs that are not approved by the guideline. Remdesivir suppresses RNA transcription by inhibiting RNA polymerase function. Chloroquine as an antimalarial drug inhibits viral cell-binding by preventing the ACE-2 receptor to be glycosylated. Both Chloroquine and Hydroxychloroquine have immunomodulatory features as well as inhibiting viral fusion because of increasing endosomal pH and preventing viral genome to be released. Azithromycin as a synergistic drug has antiviral and anti-inflammatory effects. However, neither of them has shown efficacy in lowering viral load in respiratory tracts clinically. Ivermectin as an antiparasitic drug inhibits a specific type of intracellular transporting proteins of infected cells that are used by the virus to spread infection. But, it has not shown a significant clinical advantage in trials. HIV Protease inhibitors were suggested to inhibit COVID-19 proteases but trials did not support that clinically. Nitazoxanide is an antiparasitic drug that suppresses specific enzymes of the infected cell that are hijacked by the virus for processing viral proteins. Clinical trials do not confirm that in COVID-19 patients. Different types of anti-SARS-CoV-2 monoclonal antibodies have been proved to be effective in mild to moderate cases that are at the risk of COVID severity. Some other drugs are under evaluation such as colchicine, fluvoxamine, and other immunomodulators (Covid- 19 Treatment Guidelines Panel, 2021). COVID-19 specific T lymphocytes as a plasma subset of convalescent donors have shown some progress in this field (Ferreras et al., 2021). Furthermore, RM has made some steps toward COVID-19 treatment. As previously mentioned, the efficacy of using MSCs in the treatment of the infection has been shown in different clinical studies (Sánchez-Guijo et al., 2020). However, there is still much to be done for the suggested medical, immunologic and RM treatments to be approved by valid organizations. Modeling can extensively advance this field of study.

### Stem Cell Modeling

#### Induced Pluripotent Stem Cells-Derived Models

Although embryonic stem cells (ESCs) have been used in many clinical trials and studies as a valuable source in different fields of regenerative medicine, downsides such as ethical concerns and immune rejection following allogeneic transplantation led to the development of iPSCs. These stem cells are produced by reprogramming adult somatic cells and like ESCs, can differentiate into any type of somatic cells. IPSC discovery resulted in enormous progress in research areas such as biomedicine, drug discovery, diseases pathophysiology and etiology, cell therapy, and generally, regenerative and personalized medicine (Doss and Sachinidis, 2019). Moreover, hiPSCs have been recently used as beneficial models of infectious diseases. For instance, hiPSC-derived hepatocytes have been infected with hepatitis B virus, and hiPSC-derived cardiomyocytes have been studied for cardiomyopathy of Chagas disease. Another example is the study of hiPSC-derived neural progenitor cells (NPCs) infection with Zika virus and Herpes Simplex virus-1(HSV1). Accordingly, different types of cells derived from iPSCs have been investigated for SARS-CoV-2 infection (Nolasco et al., 2020). Type2 alveolar epithelial cells (AT2s) play a crucial role in the lung by producing surfactant and differentiating into type 1 alveolar epithelial cells (AT1s). However, their proliferation capacity is poor in *in vitro* cultures. On the other hand, hiPSC-derived AT2s have greater proliferative potential. AT2s derived from hiPSCs (either in an organoid form or *in vitro* culture at the air-liquid interface) have provided a valuable tool for studying and modeling the effects of SARS-CoV-2 infection on these cells and presenting the changes in cellular and molecular mechanisms including loss of surfactant gene expression, cellular toxicity and stress, and viral entry via TMPRSS2 (Anderson and Francis, 2018; Abo et al., 2020; Nolasco et al., 2020). In addition to lung infection, SARS-CoV-2 can cause systemic inflammation and infect other systems and organs like CNS, digestive tract, and liver (Kase and Okano, 2020; Simoneau and Ott, 2020). One of the proposed mechanisms of viral migration is endothelium infection. Although direct infection of endothelial cells by SARS-CoV-2 is reported, there are controversial results from studying hiPSC-derived endothelial cells. Results of these studies showed that the infection of these cells by SARS-CoV-2 pseudo virus entry was lower than other cells like cardiomyocytes and even no infection was detected while they expressed ACE2 (Nolasco et al., 2020; Yang et al., 2020). Therefore, one of the hypotheses for the actual pathogenesis is *in vivo* up-regulation of ACE2 in response to systemic changes and interferon stimulation, as ACE2 is an interferon-stimulated gene (ISG). This hypothesis needs to be tested by models of endothelial cells from iPSCs. Besides, COVID-19 is known to bring a hypercoagulable state upon patients-probably not in a similar way to other infections- presumably by cytokine storm and endothelial dysfunction. Therefore, hiPSC-derived endothelial cells can help understand the possible novel mechanisms of hypercoagulation in this vascular-thrombotic disease (Nolasco et al., 2020). There have been concerns about cardiac manifestations of COVID-19 since myocardial injury and arrhythmias are reported in some patients. ACE2 is expressed in cardiomyocytes that makes them susceptible to SARS-CoV-2 infection. It is found that patients with heart failure exhibit more ACE2 expression that can make them more prone to severe conditions. Also, high levels of highly sensitive Troponin-I (hs-cTnI) lead to a poor prognosis of the disease. It is not completely clear whether all cardiovascular changes are directly due to the virus infection or are results of other organ dysfunctions like impaired pulmonary function. Accordingly, different studies have used cardiomyocytes derived from hiPSCs and hESCs for modeling this condition and drug screening (e.g., ACE inhibitors or ARBs, remdesivir, and chloroquine). It is found that the cells are susceptible to viral infection and replication that causes apoptosis and contractility alterations, including ceased beating. In addition, the infection induces changes in gene expression like down-regulating ACE2 expression and the genes associated with mitochondrial function (Choi et al., 2020; Nolasco et al., 2020; Sharma et al., 2020). ACE2 has well-known protective effects for the heart by inhibiting overload of angiotensin II. Studies have shown that SARS-CoV-2 causes translocation of ACE2, which results in its suppression, and also the virus increases brain natriuretic peptide (BNP) expression. Hence, the infection results in dysregulation of angiotensin balance and leads to inflammation and thrombosis as well as increasing some inflammatory cytokines. It can be concluded that both the direct cytopathic effect and the immune response of the host are responsible for the cardiac injury (Wong et al., 2020). Different neurological disorders in patients with COVID-19 (especially those with comorbidities) are reported such as headache, loss of smell and taste, meningitis, and acute hemorrhagic necrotizing encephalopathy (Kase and Okano, 2020; Moriguchi et al., 2020; Poyiadji et al., 2020; Simoneau and Ott, 2020). It seems that SARS-CoV-2 can have a direct pathogenic effect on CNS cells in addition to causing cytokine storms, but the exact mechanism remains unclear. Therefore, many iPSC-derived models have been used to understand this mechanism (Simoneau and Ott, 2020). Cysteine-rich protein 61(Cyr61) or CCN family number 1(CCN1) is a virulent factor that is known to be enhanced in SARS-COV-2 infection. Further, ACE2 expression is high in some parts of the brain like the thalamus and choroid plexus. To investigate the potential role of these receptors for CNS infection, the expression of ACE2 and CCN1 was examined in neural stem cells (NSCs) and NPCs derived from hiPSCs. The expression of these SARS-CoV-2 targets not only was seen in hiPCS-derived NSC/NPCs but also was found in the young neurons differentiated from these cells, thus SARS-CoV-2 can infect them. Moreover, this COVID-19 model was used to find a therapeutic approach. Pretreatment of hiPSCs-derived NSC/NPCs (neurosphere) with γ-secretase inhibitors (GSIs), DAPT, and compound 34, which inhibit signaling, was performed. The expression of CCN1 was significantly suppressed in neurospheres. Hence, GSIs can be used to treat CNS disorders of COVID-19, though further investigation is required (Kase and Okano, 2020). Some other studies of iPSC-derived brain organoids are addressed in the next section.

#### Other Stem Cell Models

In addition to the iPSC-derived models discussed, we reviewed models derived from other types of stem cells. For instance, scientists have used cultured human airway basal stem cells (ABSCs) as a MSC-based model of SARS-CoV-2 infection (Purkayastha et al., 2020). It was confirmed that the viral load in ABSCs that were exposed to cigarette smoking (CS) was about 2-3 folds higher than in the mock-exposed group. The result changed after 72 h due to cell apoptosis as well as patients’ genetic differences. Other effects of CS on the respiratory tract are decreased ciliated cells as well as the increased level of ABSCs as a part of a repair response stimulated by the specific exposure. None of them would happen in a normal process of virus pathogenicity due to repair mechanism inhibition. An increased rate of apoptosis is also another finding during SARS-CoV-2 infection that is confirmed by ABSCs model. This process is reinforced by CS. This model also can be applied to investigate the effect of interferon therapy on innate immune system activity state. Based on the findings of ABSC models, down-regulation of gene expression including those associated with immune responses and metabolic processes would be another result of the infection, however, genes involved in interferon signaling and chromatin organization seem to be the exceptions. Altogether, ABSCs can be a suitable MSC model in order to study the virus mechanism of action leading to acute lung injury in humans, on which the effect of environmental factors such as CS can be studied (Purkayastha et al., 2020).

### Organoid Models

Regenerative medicine has provided organoids that have many advantages in the field of disease modeling in comparison to *in vivo* and other *in vitro* models. Organoids can be generated either from adult stem cells or PSCs. Establishing organoids from PSCs like iPSCs and ESCs needs the media containing growth factors for culturing in a way that mimics the process of developing a particular structure in an embryo. On the other hand, organoids derived from adult stem cells are formed by providing a 3D matrix and growth factors for the resident stem cells of our targeted tissue (Atala et al., 2020; van der Vaart et al., 2021). This novel technology of artificially developed 3D structures is more accessible and a faster tool than animal models. Moreover, they mimic the relevant niche and maintain the genetic profile and physiological characteristics of their original tissue. Hence, since organoids have contributed to gaining more insight into the pathophysiology of different organ dysfunctions and investigating therapeutic approaches, they can serve as valuable models for investigating the SARS-CoV-2 infection and its treatment strategies (Atala et al., 2020; Mahalingam et al., 2021).

#### Lung

The primary cause of mortality in this pandemic is lung disease; therefore respiratory models including airway and alveolar organoids were used to study SARS-CoV-2. As an example of airway organoids, Lamers et al. generated 2D bronchoalveolar-like organoids in the air-liquid system and small airway 2D cultures from human small airway stem cells. The bronchoalveolar model included alveolar, basal, and rare neuroendocrine cells which were grown from 3D lung bud tip progenitor organoids. The models were infected by the virus and AT2 like cells were targeted in bronchoalveolar type while in small airway model, ciliated cells were known as the main targets of SARS-CoV-2. Moreover, to test drug screening, the bronchoalveolar model was treated with IFN-λ1. The results showed reduced viral replication and infection. Despite the advantages of studying the pathogenesis using the airway organoids, establishing more specialized alveolar systems are essential (Salahudeen et al., 2020; Lamers et al., 2021; van der Vaart et al., 2021). ARDS is one of the most severe clinical presentations of COVID-19. As previously described, AT2s have a pivotal role in lung involvement and ARDS. However, modeling AT2s *in vitro* has been challenging due to limitations like rapid dedifferentiation, loss of phenotype, and requirement of supporting fibroblasts (Huang et al., 2020; Katsura et al., 2020). Accordingly, some recent studies have developed new models of AT2s derived from iPSCs. Huang et al. generated iPSC-derived AT2s cultured in 2D air-liquid interface and 3D epithelial spheres expressing surfactant protein-C (SFTPC). The 2D cultures were used to adapt the model with the physiopathology of SARS-CoV-2 infection as it happens at the apical membrane of the cells, 2D cultures can simulate the mechanism more accurately than the 3D types. ACE2 and TMPRSS2 expression was confirmed by immunofluorescence staining and the SARS-CoV-2 infection was indicated by localizing the viral particles in different spaces like the lamellar body and tubular myelin. After the infection, the epithelial-intrinsic innate immune response including inflammatory phenotype and NF-κB signaling (1day post-infection), decreased expression of surfactant gene, cellular stress and toxicity, moderate IFN responses, and iPSC-derived AT2 death (4 days post-infection) was observed. Moreover, camostat mesylate (aTMPRSS2 inhibitor) and remdesivir administration resulted in reduced infection, which indicates therapeutic potentials (Huang et al., 2020). Katsura et al. (2020) established a novel alveosphere culture for AT2s from primary lung tissue that expressed ACE2 and TMPRSS2, and was permissive to the infection. Similar findings were reported after the SARS-CoV-2 infection including up-regulation of inflammatory signaling, cell death, surfactant loss, and the IFN response. In addition, pre-treatment of the alveospheres with IFNs revealed a prophylactic effect and reduced viral titers. One of the differences between these two studies is the time of IFN response; in the alveosphere model, the IFN pathway was detected 48 h post-infection while in the iPSC-derived model, it occurred 4 days after the infection. Another 3D culture technique was developed for hAT2s from healthy donor lungs by Youk et al., which established the cellular polarity and made the AT2s stable, although it did not completely present the full alveoli. It demonstrated transcriptional changes after SARS-CoV-2 infection, IFN response, and ISGs expression at 3 days post-infection (Youk et al., 2020). By comparing the details and results of the mentioned studies, some models have considerable advantages. For instance, 2D structures showed more virus titer than the 3D organoids, which can be more useful for testing the antiviral agents. Further, in 3D organoids, the apical side of the cells is inside the model while in the 2D culture, especially the bronchoalveolar-like model, they are exposed to the air. Hence, they are suggested to be more relevant and suitable for studying the virus pathogenesis (Lamers et al., 2021). Besides, Han et al. generated an *in vivo* model of lung organoids. The organoids were developed from human pluripotent stem cells (hPSCs) and the presence of AT2-like cells and the expression of ACE2, and TMPRSS2 were validated. To make the *in vivo* model, progenitor cells of the lung were transplanted subcutaneously into non-obese diabetic (NOD) severe combined immune deficiency (SCID) gamma (NSG) iL2RG^*nul*^ mice and produced structures with AT2-like cells expressing ACE2. To test the infection, the xenograft was inoculated with SARS-CoV-2 entry virus. The RNA analysis confirmed the infection, and the infected organoids showed enhanced chemokine signaling compared to the mock-infected group. Moreover, the inhibitory effects of some FDA-approved drugs including imatinib, mycophenolic acid (MPA), quinacrine dihydrochloride (QNHC), and chloroquine were investigated and a significant reduction of infection and virus entry was observed. In addition, the transplanted mice were treated with imatinib mesylate, MPA, or QNHC before virus inoculation that decreased the luciferase staining. Conclusively, lung organoids were introduced as useful candidates for modeling the drug discovery of COVID-19 (Han et al., 2021).

It is evident that organoids can contribute to understanding the mechanism of this new infection. The airway models can be studied for virus shedding and mild forms of the disease and the alveolar models are more relevant for research on severe stages and complications of COVID-19 (van der Vaart et al., 2021).

#### Liver

A liver organoid is produced by differentiation of hiPSCs into definitive endoderm and then, inducing liver cells (mainly albumin^+^cells). The immunostaining showed ACE2 expression in most albumin^+^ hepatocytes. Next, using the SARS-CoV-2 pseudo-entry virus showed that the organoid is permissive to the infection that is similar to adult primary hepatocyte and cholangiocyte organoids. The results were consistent with the reports of COVID-19-related hepatitis, which was observed in some patients (Yang et al., 2020; Yu et al., 2020). In order to investigate the pathogenesis of SARS-CoV-2 infection in the liver, human liver ductal organoids were generated from liver bile duct-derived progenitor cells in a long-term 3D culture system. The immunostaining indicated ACE2^+^ and TMPRSS2^+^ cholangiocytes and the examination of SARS-CoV-2 genomic RNAs showed susceptibility of the cells to infection and increased viral load. Besides, the significant decrease in the viral load 24 h after the infection was due to enhanced expression of apoptotic factors and cholangiocytes’ death. Since the main function of cholangiocytes is the transportation of bile acid, the tight junction between them is necessary for the collection and excretion of bile acid from hepatocytes into bile ducts. In SARS-CoV-2 infection, the expression of genes involved in cell junction organization such as claudin1 (CLDN1), a bile acid transportation like solute carrier family 10 member 2(SLC10A2) and cystic fibrosis transmembrane conductance regulator (CFTR) is decreased. Hence, it causes liver damage by impairing cholangiocyte function and accumulation of bile acid. In addition, the organoid can be used for drug discovery to prevent liver injury of COVID-19 (Zhao et al., 2020; Youhanna et al., 2021).

#### Eye

Although it is reported that eyes are affected in COVID-19, there is no available evidence of retinal involvement (Ahmad Mulyadi Lai et al., 2021). ACE2 and TMPRSS2 are known to be expressed in the retinal organoid (Mahalingam et al., 2021). Accordingly, hiPSC-derived retinal organoids and a retinal monolayer culture dissociated from the organoid were utilized. Real-time quantitative PCR (qPCR) and immunofluorescence staining showed ACE2 and TMPRSS2 expression both in the organoid and monolayer cultures. Additionally, SARS-CoV-2 pseudovirus containing the GFP coding sequence was employed to study the susceptibility of the organoids and the monolayer cultures to the infection. Detecting GFP signals demonstrated that SARS-CoV-2 could infect them and enhance the expression of some genes related to the apoptosis pathway and inflammation response. On the other hand, in a pre-print study, Makovoz et al. has produced hPSC-derived whole eye organoids. The results showed that the expression of ACE2 and TMPRSS2 in the cornea was high and the corneal organoids were permissive for virus entry and infection. Limbus was found to be the most susceptible part which exhibited a significant NF-κB mediated inflammatory response. However, it is not clear whether the inflammation is due to direct infection or the virus spreading to the eye through systemic infection (Makovoz et al., 2020; Yu, 2021). Altogether, it seems that the eye is a route of infection; hence the organoids and monolayer cultures represent useful models of studying the SARS-CoV-2 mechanism of pathogenesis (Ahmad Mulyadi Lai et al., 2021).

#### Kidney

One of the most common organ damages in COVID-19 patients is AKI. Moreover, ACE2 is significantly expressed in convoluted tubules but it is not clear whether the kidney injury is due to direct cytopathic effects of the virus or immunopathogenic damage (Xia et al., 2020; Mahalingam et al., 2021). Hence, 2D conditional reprogramming (CR) and 3D organoid of human kidney proximal tubule epithelial cells (KPTECs) were established as novel platforms. The DNA repair ability and lineage function, and expression of specific function markers were maintained in CR KPTECs. Interestingly, the ACE2 expression was detected in both models with a higher level (around two-fold) in the 3D organoid. The permissiveness of the reprogrammed cells and the models for SARS-CoV-2 infection was estimated by reading relative luciferase units (RLU) after the pseudovirus infection. Hence, these cells have served as physiological platforms to model SARS-CoV-2 infection, investigate the kidney response, and use them for drug discovery (Xia et al., 2020). Further, one of the proposed treatments of COVID-19 is human recombinant soluble ACE2 (hrsACE2) which has undergone phases 1 and 2 clinical trials. The potential inhibitory effect of hrsACE2 was tested in kidney organoids developed from hESCs. A mixture of hrsACE2 and particles of SARS-CoV-2 was used for infecting the kidney organoid and qRT-PCR indicated a dose-dependent reduction in the level of SARS-CoV-2infection and viral load (Monteil et al., 2020).

#### Gut

Gastrointestinal symptoms of COVID-19 are one of the most familiar and prevalent effects of the infection that are present in around 50% of patients (Krüger et al., 2021). For improving research quality, different intestinal and colonic organoids were produced. For instance, a human small intestinal organoid (hSIO) was developed from primary gut epithelial stem cells. Cultured hSIOs were exposed to both SARS-CoV and SARS-CoV-2 and qRT-PCR assessment showed infected enterocyte lineage cells with both viruses. Further, mRNA sequence analysis indicated that SARS-COV-2 caused ISGs and cytokines induction and up-regulation of ACE2 stronger than SARS-COV (Lamers et al., 2020). In another study, human intestinal organoids were derived from PSCs. ACE2 and TMPRSS2 expression was seen in the intestinal cells except for goblet cells, which lacked ACE2 expression. The viral RNA levels exhibited infection of the organoid cells (not goblet cells). Additionally, the inhibitory effects of remdesivir, famotidine, and EK1 (a peptidic pancoronavirus fusion inhibitor) were investigated. The results showed that remdesivir blocked SARS-CoV-2 replication in a dose dependent manner; EK1 decreased the number of infected cells. However, famotidine did not show any inhibitory effect in the intestinal organoids. Thus, human intestinal organoids derived from PSCs represented a valuable physiological model for experimental studies (Krüger et al., 2021). Further studies indicated ACE2 and TMPRSS2 expression as well as SARS-COV-2 permissiveness of colonic organoid derived from hPSCs. For studying COVID-19 *in vivo*, the colon organoids were transplanted under the kidney capsule of NSG mice. In addition to ACE2 detection, infecting the organoid with SARS-CoV-2 via intra-xenograft inoculation confirmed the permissiveness and the viral infection of the organoids. Next, the inhibitory effects of some FDA-approved drugs were tested. Colonic organoids were treated with imatinib, QNHC, and MPA and then infected with SARS-CoV-2. They all resulted in decreased viral RNA level and nucleocapsid protein expression that means blocking the infection. Taken together, the colonic organoids have introduced an experimental model for drug discovery (Han et al., 2021).

#### Cardiovascular System

The endothelial damage of COVID-19 is a matter of great importance that needs further investigation. Not only As previously mentioned, hiPSCs derived endothelial cells express ACE2, yet they seem not to be infected with SARS-CoV-2 since the pseudo-entry virus resulted in low luciferase activity (Yang et al., 2020; Yiangou et al., 2020). On the other hand, capillary organoids derived from iPSCs exhibited SARS-CoV-2 replication that was revealed by analyzing qRT-PCR. Moreover, as discussed in kidney organoids, Monteil et al. revealed that the infection was reduced by adding hrsACE2 (Monteil et al., 2020). Collectively, there is a possibility that the injury of the endothelium is not due to direct infection but the paracrine effects that should be studied in different relevant models (Yiangou et al., 2020).

#### Brain

In spite of the reports on finding SARS-CoV-2 in CSF and brain, the exact prevalence of CNS infection and the mechanism of virus entry and pathogenesis are not fully understood (Pellegrini et al., 2020; Song et al., 2021). In a hiPSC-derived brain organoid, SARS-CoV-2 infected the neurons and induced cell death despite low levels of ACE2 expression. However, a productive replication of the virus was not detected. Besides, SARS-CoV-2 caused aberrant Tau localization and phosphorylation. Tau is a protein that is responsible for stabilizing the microtubules of neurons and promoting axonal growth. Tau dysfunction is known to be involved in Alzheimer’s disease. While it is mainly localized at the axons, the infected neurons showed an increased level of Tau into the cell somas. Moreover, it was revealed that virus entry caused tau phosphorylation at the T231 site, which is remarkable for aberrant phosphorylation leading to neural toxicity. Although tau hyper phosphorylation and abnormalities in the infected neurons were detected, it is not completely clear whether they are the direct effects of the virus or the results of neural stress. Hence, more investigation is suggested (Ramani et al., 2020). Another study developed dorsal forebrain organoids from hESCs and reported that ACE2 was expressed in the organoid with a significant decreased level in the progenitor and stem cells. The neurons were found susceptible to the spike-containing SARS-CoV-2 pseudo-entry virus but increasing the viral load did not elevate the virus infectivity (Yi et al., 2020). Besides, the BBB and blood-CSF-barrier (B-CSF-B) are known as major obstacles to the entry of pathogens like SARS-CoV-2. B-CSF-B, which is simpler than BBB, consists of an epithelial layer of the choroid plexus, which has interactions with blood. In addition, it participates in the immune response by producing some inflammatory cytokines in the CSF. To test the choroid plexus role in SARA-CoV-2 neurotropism, the hPSC-derived brain organoids, which represented the choroid plexus and the cortical tissue, were developed. ACE2 and TRMPSS2 expression were detected in the choroid plexus and not in the neural progenitors. Next, the organoids were incubated with both SARS-CoV-2 spike pseudo virus and live SARS-CoV-2. The results showed that the epithelial cells of the choroid plexus were susceptible to the virus infection and its replication while the neural region exhibited little to no infection. Moreover, at 4 days post-infection, considerable damage to the barrier integrity and a decrease in the internal fluid (CSF leakage) were reported (Pellegrini et al., 2020). Similar findings were reported by Jacob et al. who generated monolayer cultures of neurons, astrocytes, and microglia as well as organoids of the cerebral cortex, hippocampus, hypothalamus, midbrain, and choroid plexus (Jacob et al., 2020). The results showed no infection in the monolayer culture of microglia and sparse infection in cortical neurons and astrocytes. Additionally, introducing SARS-CoV-2 to the multiple organoids indicated a limited infection rate in neurons and astrocytes, which did not exhibit a significant increase, days after the infection. Notably, the choroid plexus regions in some organoids like the hippocampus showed greater numbers of infected epithelial cells. For more investigation, hiPSCs were grown to differentiate into the choroid plexus organoid, in which a productive infection was observed. SARS-CoV-2 led to enhanced cell death, inflammation, and changes in the barrier and secretory function (Jacob et al., 2020).

**FIGURE 1:**
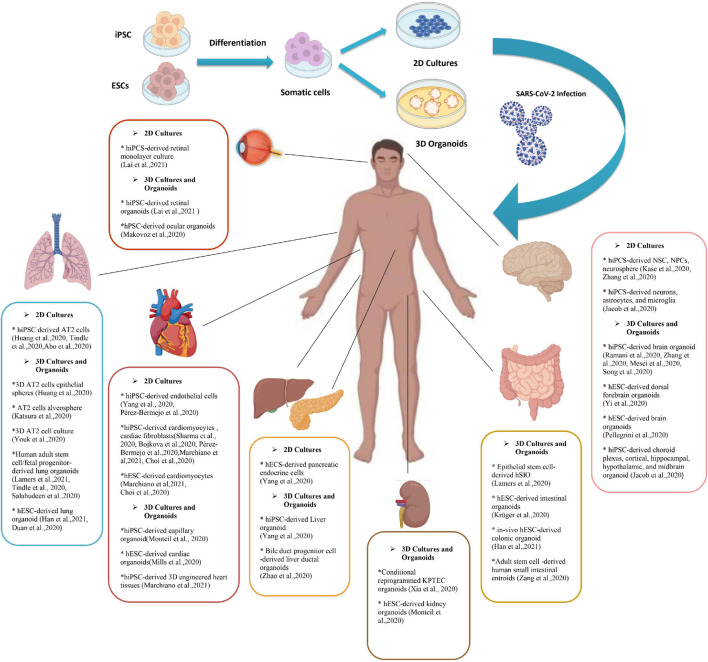
A Summary of pluripotent stem cell (PSC) derived 2d and 3D models of SARS-CoV-2 infection. Human induced pluripotent stem cells (hiPSCs) and human embryonic stem cells (hESCs) have been used to produce adult somatic cells of different organs. Culturing these cells can become either 2D models of the cells or tissue or the novel model of 3D organoids. Various studies have used PSCs (and some adult stem cells) to investigate the SARS-CoV-2 infection mechanism and its manifestations. This figure provides a brief review of currently available researches on different stem cell-based models to discover the SARS-CoV-2 mechanism of action and potential therapeutic strategies. PSCs, Pluripotent stem cell; hESCs, Human embryonic stem cells; hiPSCs, Human induced pluripotent stem cells; SARS-CoV-2, Severe acute respiratory syndrome Coronavirus-2; NSCs, Neural stem cells; NPCs, Neural progenitor cells; AT2s, Type2 alveolar epithelial; 2/3-D, 2/3 dimensional; KPTECs, Kidney proximal tubule epithelial cells; hSIO, Human small intestinal organoid.

The number of studies using iPSC-derived cells and organoid models of COVID-19 associated disorders is rising. Most of these studies are discussed in the text and some other researches and pre-print literature are depicted in Figure 1. Also brief data on the pros and cons of each stem cell or organoid model check Table 1.

**TABLE 1:** Pros and cons of stem cell and organoid models of Severe Acute Respiratory Syndrome Coronavirus-2 (SARS-CoV-2).

Type of model		Pros	Cons	References
Stem cell models	MSC-based models	- Lower cost in contrast to lung organoid models	- Various differentiation capacity of stem cells from different donors - variation in the number of infected cells in each set of cultures - Limited sample size due to the challenges and costs of obtaining human tissue - Lack of inflammatory cells in the cultures - Offering only short-term culture capabilities in contrast to the stability and indefinite growing capabilities of organoid models	Purkayastha et al., 2020; Chugh et al., 2021
	IPSC-derived models	- Generating various differentiated cells with same genetic background -Mimicking the biology of host-virus interaction - Patient (genotype)-specific - Suitable for gene editing - The susceptibility to become more complex via co-culturing - Easy to keep in culture and maintain	- Labor/time consuming and higher cost culture - Possible variations among paths of differentiations - Some limitations in representing the exact *in vivo* manifestation of infected tissues	Nolasco et al., 2020; Chugh et al., 2021
Organoid models	Brain	- Allowing the scientist to explore neurotoxic effect of COVID-19 - A suitable platform to study the antiviral effects of anti COVID-19 drugs through preserving BBB from viral damage	-Absence of vascularization as in human brain - Requiring modification for making long-term observation possible	Chugh et al., 2021
	Gut	- Containing all proliferative and differentiated cell types of the *in vivo* epithelium - Reflecting the high susceptibility of gastrointestinal system to be infected *in vivo*, by representing the high rate of enterocytes infection as the most common cell type pf intestinal system.	- Weakness in showing the patient’s defense mechanisms against digestive system infection during COVID-19 (intestinal flora and lymphatic system)	Lamers et al., 2020; Yu, 2021
	Kidney	- Effectively representing COVID-19 associated AKI - Showing the efficacy of combination therapy using Remdesivir with human recombinant soluble ACE2 in reducing virus entry and replication.	- Cannot mimic the exact features of native renal tissue	Chugh et al., 2021
	Cardiovascular system	- Suitable for exploring the efficacy of new drug candidates in reversing cardio-toxic effects of the virus - Appropriate for validating the potential role of specific genetic variants in COVID-19 pathology	- The impossibility of mimicking arrhythmia and myocardial infarction - The need for careful adjusting of drug doses because of higher drug levels in the organoid in comparison to human blood due to the absence of metabolic organs in the cardiovascular organoids	Yiangou et al., 2020; Yu, 2021
	Lung	- Allowing efficient viral replication - Suitable for exploring interactions between human cells and viruses and the response of lung stem cells to SARS-CoV-2 - Can be used to test drugs targeting a wide range of viruses	- The absence of stroma and immune cells - The absence of a definite culturing protocol in order to prevent bias	Chugh et al., 2021; Lamers et al., 2021; Mallapaty, 2021; Yu, 2021
	Liver	- Investigated liver tissue damage of SARS-CoV-2 *ex vivo*. - Mimicking host-virus interaction due to retaining the biology of individual tissues such as preserving human-specific ACE2 + /TMPRSS2 + population of cholangiocytes	- Inability to reflect the cellular complexity of human hepatobiliary system for instance specific immune cell subsets -Lack of non-parenchymal cells -Presenting immature liver phenotype	Zhao et al., 2020; Lui et al., 2021; Youhanna et al., 2021; Yu, 2021

*SARS-CoV-2, severe acute respiratory syndrome coronavirus 2; AKI, Acute kidney injury; MSC, Mesenchymal Stem Cell; IPSC, Induced pluripotent stem cells; BBB, Blood Brain Barrier; ACE2, Angiotensin-converting enzyme 2; TMPRSS2, Transmembrane protease; serine 2.*

### Animal Models

**FIGURE 2:**
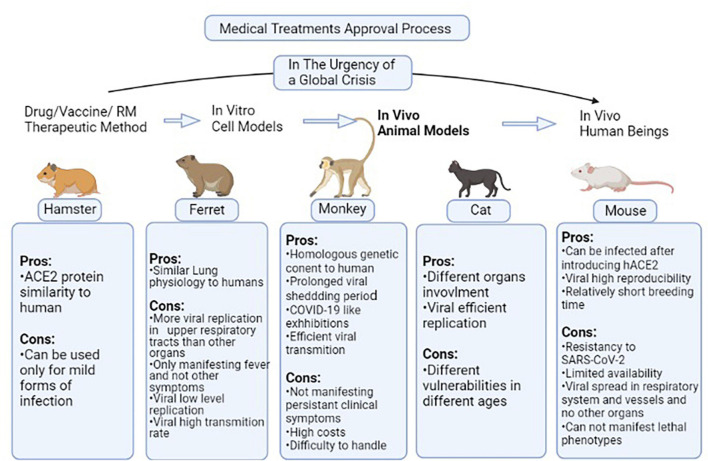
Pros and cons of each species model discussed in Table 1. In non-critical situations, the routine process of a medical treatment to be approved begins from *in vitro* level of experiment and passes toward human experiments through passing the animal model testing stage. In the situation of global crisis such as the COVID-19 pandemic, the *in vitro* and animal model testing stages are not much focused on and the candidate therapeutic methods are directly applied to volunteers. But this gap has to be filled as soon as possible in order to reduce the drug/vaccines/Regenerative Medicine (RM) therapies rate of error. This figure provides the pros and cons of different species models in the case of studying animal models used in this field (Kumar et al., 2020; Rahim and Muhammad, 2020; Zheng et al., 2021). ACE2, Angiotensin-converting enzyme 2; hACE2, human-ACE2; SARS-COV2, Severe acute respiratory syndrome coronavirus 2; RM, Regenerative Medicine.

As previously discussed, COVID-19 is a new scientific field of study with noticeable gaps that have to be covered especially in testing the efficacy of new therapeutic methods in the field of clinical as well as regenerative medicine. Several animal models can be used with similarities to the human body in some aspects. These species also have been used for testing drug/vaccine candidates of clinical medicine and proved their efficacy, so it is implied that they can be suitable models for regenerative medicine investigations. Different species have been used in this field among which, mice are the most common (Sun et al., 2020). The problem is that mice are resistant to SARS-CoV-2 therefore this issue is resolved by introducing the human ACE2 (hACE2) receptor via an adenovirus into the cells (Atala et al., 2020; Zheng et al., 2021). In a study, cytokeratin 18 (KRT18) promoter, which is an epithelium specific gene, was used for the host cells to express hACE2 (Chow et al., 1997; Zheng et al., 2021). This process causes pneumonia along with weight loss, severe pulmonary pathology, and SARS-CoV-2 replication in the lungs (Sun et al., 2020; Zheng et al., 2021). In some other studies, Lipopolysaccharide has been used to simulate the hyperinflammatory state caused by SARS-COV-2 infection (Molinaro et al., 2020). Mice-based studies confirmed the role of the interferon I (IFN-I) pathway in the clearance of virus as well as the role of interferon II (IFN-II) signaling through signal transducer and activator of transcription 1 (STAT-1) in diminishing clinical severity and hyperinflammatory state of the respiratory system (Sun et al., 2020). In an experiment of Ad5-hACE2 mice, the application of plasma from recovered patients improved the disease severity in addition to increasing the clearance rate of the virus as well as remdesivir (Sun et al., 2020). Leukosomes have been suggested to promote the efficacy of anti-inflammation drugs when applied as a drug delivery system (Molinaro et al., 2020). Ferret is another animal model of SARS-COV-2 infection, which is prone to SARS-COV *per se* and presents symptoms like cough, rhinorrhea, and lower physical activity (ter Meulen et al., 2004; Park et al., 2020). Positive samples of infectious virus only could be collected through upper respiratory tract washes, and not other collected specimens such as a rectal swab, which was positive of low copy numbers of non-infectious virus (Shi et al., 2020). Then, the application of suggested drugs such as lopinavir-ritonavir, Hydroxychloroquine (HCQ), emtricitabine-tenofovir, and human monoclonal antibodies led to lower severity of clinical presentations (ter Meulen et al., 2004; Park et al., 2020). Another study showed that as well as ferrets, cats, and with less susceptibility, dogs are prone to SARS-CoV-2. In a study using cats as animal models, it was proved that the infection transmits in an airborne state among cats (Shi et al., 2020). Besides, in a study of Syrian hamsters as one of the permissive species to SARS-CoV-2, the STAT-2 signaling mechanism has been considered as one of the mechanisms involved in the virus pathogenesis as well as its protective role as part of the immune system (Boudewijns et al., 2020). Each discussed model has some advantages that make it appropriate to be used as a human body simulator but some disadvantages that make investigations more challenging. However, assessing these disadvantages opens new doors toward developing more efficient animal models. Several types of species models, specific manifestations of each after virus inoculation, and results of suggested drugs/vaccine candidates have been provided in Table 2. Also, pros and cons of each species model are accessible in the Figure 2.

**TABLE 2:** Animal models of Severe Acute Respiratory Syndrome Coronavirus-2 (SARS-COV-2) and the results of tested drug/vaccine candidates.

Animal model	Method of model designing	Presented symptoms	Tested drugs/vaccine candidates	Results	References
Ferrets	Readily permissive to the virus	-↑Body temperatures -Infectious virus shedding through upper respiratory tract washes - Loss of appetite	-Lopinavir-ritonavir -HCQ -Emtricitabine-tenofovir	-↓Overall clinical scores than control group -↓Virus titer in emtricitabine-tenofovir-treated group	ter Meulen et al., 2004; Park et al., 2020; Shi et al., 2020
			Prophylactic human monoclonal antibody (against SARS-CoV)	-↓Viral replication in the lung -↓Viral induced lung pathology	
Outbred domestic cats	Readily permissive to the virus	-Infectious virus shedding through nasal turbinate, soft palates, tonsils, tracheas, lungs -Massive lesions in the nasal and tracheal mucosa epitheliums and lungs			Shi et al., 2020
hACE2 BALB/c mice	-Introducing hACE2 via adenovirus -Introducing KRT18	-Severe pneumonia -vasculitis -Severe brain involvement in some cases -Anosmia -Weight loss	-Plasma of recovered patients -Poly I:C -Remdesivir	-Protection against lethal severity -Accelerating virus clearance	Sun et al., 2020; Zheng et al., 2021
BALB/c mice	Intraperitoneal injection of LPS	Severe lung damage (ARDS)	DEX-loaded leukosomes	-↑Therapeutic activity of dexamethasone -↓Inflammatory response	Molinaro et al., 2020; Zhang N. N. et al., 2020; Kaspi et al., 2021
			ARCoV vaccine candidate	-↑Innate immune cells in the muscles in IM injection -↑Neutralizing antibodies and IgG -↑Cellular immunity (CD4 + or8 + T cells) -↑IFN-II, TNF-a, IL-2 -NC in IL-4 and IL-6	
			Exo MSC-NTF	-↓Alveolar wall thickness, fibrin presence, and neutrophil accumulation -↑Oxygenation saturation -↓Proinflammatory cytokines (IFN-II, IL-6, TNF-a, RANTES) - ↓ TF, TAT	
C57BL/6 mice (Ifnar1-/- or Il28r-/-)		- Mild lung pathology (both) -↑Viral replication (Ifnar1-/-) -↑Intra-alveolar hemorrhage, peribronchiolar inflammation (Ifnar1-/-)	Plasma of recovered patients	-3-10-fold↓in viral loads -NC in histopathological features -↓Akt1 mRNA levels -↑DDX58, cGAS mRNA levels	Yang et al., 2020
Immunocompetent BALB/c mice	Inoculated with MASCp6 virus	-Moderate pneumonia and inflammatory responses in the lung and trachea	ARCoV vaccine candidate	-Full immunization	Zhang N. N. et al., 2020
Macaca fascicularis	Readily permissive to the virus		ARCoV vaccine candidate	-↑IgG -↑IFN-II -↑IL-4 + /CD4 + cell response(no difference between vaccinated and placebo group)	Zhang N. N. et al., 2020
Syrian hamsters (WT)	Readily permissive to the virus	-Bronchopneumonia and inflammatory response, neutrophil infiltration, multifocal necrotizing bronchiolitis and edema in the lungs	-VHH-72-Fc -Plasma of recovered patients	-↓Viral loads in the lung (VHH-72-Fc)	Boudewijns et al., 2020
Syrian hamsters (STAT2-/- or IL28R-a-/-)	Readily permissive to the virus	-↓ISG expression -↓Antiviral response -NC in viral RNA levels in the lung (more infectious virus in STAT2-/-) -↑Proinflammatory cytokines in the lung -↓Lung pathology and PMN cells infiltration (STAT2-/-) -Bronchopneumonia and peribronchiolar inflammation (IL28R-a-/-)			Yang et al., 2020

*hACE2, human Angiotensin-Converting Enzyme 2; KRT18, cytokeratin 18; HCQ, Hydroxychloroquine; LPS, lipopolysaccharide; DEX, dexamethasone; ARCoV, a vaccine candidate with the component of a mRNA sequence encoding the receptor binding domain of the virus which is encapsulated in lipid nanoparticles; IFN-II, interferon II; TNF-a, tumor necrosis factor alpha; IL-2, interleukin-2; NC, no change; Exo MSC-NTF, MCS-derived exosomes producing neurotrophic factor; RANTES, CCL5; TF, Tissue Factor; TAT, Thrombin Antithrombin complex; MASCp6, mouse-adapted strain at passage 6; Ifnar1-/- mice, C57BL/6 mice with a genetic ablation of their type I interferon receptors; Il28r-/- mice, C57BL/6 mice with a genetic ablation of their type III interferon receptors; WT, wild type; STAT2-/- hamster, lacking type I and III IFN signaling; IL28R hamster, lacking IFN type III signaling; ISG, interferon-stimulated genes; GI, gastrointestinal; PMN, polymorphonuclear; VHH-72-Fc, SARS-CoV-2- specific single-domain antibody Fc fusion construct.*

### Challenges and Limitations

Despite all advantages of the models which were discussed, there are different challenges in the experimental researches on SARS-CoV-2 infection. IPSCs have been widely studied especially for generating organoids, to model the new pandemic disease, but there are some limitations that should be mentioned. For instance, there are some reports that during the reprogramming process, some of the epigenetic memory of donor cells is retained in iPSCs that can result in biased differentiation to target cells. Moreover, there are risks of tumorigenicity in the case of cell transplantation which are contaminated with undifferentiated iPSCs. However, novel protocols for differentiation and the purification process as well as using molecules that cause selective death of these cells have decreased the risks but more follow-up is needed. Besides, differentiating somatic cells from hiPSCs is considered to be expensive and time-consuming (Doss and Sachinidis, 2019; Nolasco et al., 2020). Notable bottlenecks of modeling using iPSC-derived cells are lack of maturity and exhibiting embryonic-like characteristics, especially in late-onset diseases. Some proposed ways to overcome these drawbacks are treatment with mitochondrial stress inducers to provoke aging and direct differentiation of somatic cells like fibroblasts to the relevant cells like neurons that share similar cell markers. On the other hand, novel technological advances like gene editing, organ-on-chip, and “-omics” methodologies can improve the iPSC-based therapeutic approaches (Doss and Sachinidis, 2019). Organoids, as a product of advancements in the fields of PSCs and cell therapy, ECM biology, and tissue engineering, have played a critical role in experimental studies. In the field of RM, organoids have been presented for transplantation instead of organs like the liver, as they recapitulate the main aspects of the organs. Moreover, they represent a step forward to drug screening and a promising tool for personalized medicine. However, there are some obstacles both in the future transplant process and the current modeling area of this novel tool. Some of the challenges and disadvantages of different organoids and iPSC-derived cells are addressed in Table 1. The shape of the organoids, their size, and cell composition and maturation are not completely under control. Additionally, modeling complex and chronic diseases needs a suitable microenvironment and is based on advanced technology in bioengineering (Prior et al., 2019). Major limitations of organoids in studying SARS-CoV-2 infection include their simplicity and lack of immune cells. Therefore, the studies mainly focus on intrinsic pathways. For instance, SARS-CoV-2 causes epithelial damage and inflammatory response in the lung. Hence, the absence of immune cells and stroma are known as major challenges and limitations of lung organoids (Chugh et al., 2021). It is suggested that the organoids can be introduced to immune cells in culture to investigate extrinsic mechanisms of actions (Katsura et al., 2020; Yang et al., 2020; Youk et al., 2020). Accordingly, hESCs were utilized in a study which is pre-print at the time of writing this review, to generate a co-culture of lung cells and macrophages. The results showed a reduction of SARS-CoV-2 N protein expression after adding macrophages, though N protein was detected in the macrophages *per se*. More studies are required to find the exact role of macrophages, and also other parts of the immune system in the pathogenesis of this virus (Duan et al., 2020; Simoneau and Ott, 2020). In another pre-print study, a mixture of the upper airway and alveolar cells derived from hiPSCs was developed. It showed that the airway cells are more prone to infection than alveolar cells in a manner that was similar to the patients’ lungs. Further, these novel models can be served as beneficial platforms for drug discovery (Simoneau and Ott, 2020; Tindle et al., 2021). In addition, since endothelial dysfunction and vascular impairment like thrombosis and microangiopathy have been found in patients’ lungs, adding endothelial cells to lung organoids may lead to generating more relevant models (Lamers et al., 2021). Due to the complicated behavior of SARS-CoV-2, the involvement of multiple organs, and their interactions with different types of cells and stem cells, more advanced technologies like organ-on chips or complex organoids can be more beneficial (Chugh et al., 2021). Among challenges in brain organoids, the absence of vasculature in choroid plexus organoids and the presence of not fully mature neurons in cortical organoids, which affects the susceptibility to infection, would be worth mentioning (Pellegrini et al., 2020). Moreover, the results of studying SARS-CoV-2 infection in astrocytes, microglia, and cortical neurons are controversial and to some extent, inconsistent. Since these cells have a critical role in CNS homeostasis, developing brain organoids containing these kinds of cells could be useful to take a step forward (Ramani et al., 2021). Besides, the current technology of organoids cannot manifest the communication between different organs and immunological responses in COVID-19 infection. In this context, animal models are more advantageous (Yu, 2021). As one of the most necessary major phases in manufacturing biomedical products, the ideal animal models should be able to simulate the disease in humans and its manifestations as similar to humans as it is possible. For COVID-19 modeling, the animal model must be permissive to the infection. Transgenic mice that express hACE2 are one of the best animal models, yet their availability is limited. Another mouse model is the adeno-associated virus delivery-based model, which expresses ACE2 in non-relevant cells and makes the interpretation of the analyzed data quite hard. Hamsters can model SARS-CoV-2 infection but they can only be used to study the mild forms of the disease because the lung pathology resolves to normal after 14 days post-infection (Kumar et al., 2020). As it was mentioned before, an important point about modeling this disease is that additional risk factors like advanced age and chronic diseases such as diabetes and obesity are associated with increased morbidity and mortality. These comorbidities cause alterations including the dysregulation in body homeostasis and repair mechanisms that make the infection progress to its severe and lethal forms. Hence, developing models with mentioned features to provide a platform for shifting the disease from a curable infection to a multi-organ failure state and ARDS can be beneficial. Accordingly, the results of using aged animals such as aged mice and old macaques showed higher levels of lung injury and inflammation. Other modified models for obesity (e.g., ob/ob and high-fat diet-induced obesity mice), diabetes (e.g., STZ-treated and NOD mice), and immune deficiency/impairment (e.g., SCID and STAT1-knockout mice) might be more easily available to analyze the progression of the severe status of SARS-CoV-2 infection (Croy et al., 2007; Cleary et al., 2020; Payab et al., 2021). Another advantage of using proposed animal models is the possible ability to test the efficacy of interventions and treatments in different stages of the disease. When animal models which can develop multi-organ failure are used, suggested therapies for the mild to severe conditions of the infection can be tested in a pre-clinical setting. Therefore, the safety issues of the drugs and even prophylactic agents might be explored better (Cleary et al., 2020). Collectively, an appropriate model which develops the disease, the body response, and the comorbidities for a comprehensive understanding of the pathogenesis and possible treatments is still elusive. Though, available models have led to invaluable information on the disease and different vaccines (Cleary et al., 2020; Kumar et al., 2020).

## Conclusion and Closing Remarks

The COVID-19 pandemic has placed a huge burden on people, communities, and health facilities all around the world. Great attempts toward understanding the disease and finding treatments and vaccines to fight against SARS-COV-2 are being made. Giant clinical trials such as the world health organization (WHO)’s Solidarity clinical trial and the randomized evaluation of COVID-19 (RECOVERY) trial of the United Kingdom are global platforms that are trying to identify the treatments of SARS-CoV-2 infection (No Author, 2020). Current available approved drugs for treating patients with COVID-19 are remdesivir, dexamethasone, and immunomodulators including tocilizumab and sarilumab (IL-6 inhibitors), baricitinib (a Janus kinase (JAK) inhibitor), for hospitalized patients, and anti-SARS-CoV-2 monoclonal antibodies like sotrovimab and casirivimab for non-hospitalized adults. In addition to these drugs, RM-based approaches including transplantation of progenitor or stem cells, tissues, and exosomes have also gained attention. Several trials of cell therapy are investigating the potential therapeutic effects of stem cells (Asgharzade et al., 2021; Covid-19 Treatment Guidelines Panel,2021; Rodriguez-Guerra et al., 2021). Besides, RM has been considered to be a promising field for drug and vaccine discovery and exploring the pathogenesis of the virus through disease modeling. Stem cells (especially iPSCs) and organoids, as major components of RM, have been recognized as useful models of SARS-CoV-2 infection. Various PSC-derived cell types (e.g., AT2s, cardiomyocytes, and neurons) were generated to study the SARS-CoV-2 effect on human organs and systems. Regarding the extensive data to support the value and benefits of hiPSC-derived somatic cells to model different diseases as well as various kinds of viruses, several studies on SARS-CoV-2 infection have used this RM-based model. Developing hiPSC-derived AT2s has clarified some aspects of the viral pathogenesis in the lungs. Moreover, endothelial cells derived from hiPSCs are known as a practical tool for studying the hypercoagulable state, which is caused by SARS-CoV-2 (Atala et al., 2020; Nolasco et al., 2020). The unique ability of SARS-CoV-2 to cause myocardial injury in a considerable percentage of patients has inspired scientists to explore the pathogenic mechanism. Research on hiPSCs has revealed that the virus directly affects the cardiomyocytes and actively replicates in these cells. Additionally, as mentioned before, the virus causes down-regulation of ACE2 expression, in contrast to other tissues like the intestine (Lamers et al., 2020; Wong et al., 2020). In addition, the antiviral effect of remdesivir and chloroquine was assessed in cardiomyocytes derived from hiPSCs and hESCs. Remdesivir exhibited stronger antiviral activity than chloroquine. However, it induced cardiotoxicity by causing QT prolongation and reducing cell viability at a higher concentration than the estimated peak plasma concentration. The results not only introduce the cardiomyocytes derived from hPSCs as a powerful experimental model for drug screening but also advise close monitoring of patients undergoing treatment with remdesivir, especially those with chronic heart diseases or severe form of COVID-19 (Choi et al., 2020). As well as hPSCs, organoids have acted as both *ex vivo* and *in vivo* models for lung injury in COVID-19. Transplantation of lung organoids into NSG mice and testing some proposed drugs have made the models more realistic and experimental (Han et al., 2021). Other organoid models including small and large intestine, kidney, retina, liver, pancreas, heart, and brain organoids are used and discussed in this review. For example, brain organoids and iPSC-derived neural cells have revealed some insights into the neurotropism of SARS-CoV-2. The number of these studies is still limited and the results are controversial, but they have been helpful in finding some of the underlying causes of neurological defects and also, testing some drugs. Based on currently available data, infecting the choroid plexus is a major component of brain damage. Different studies have suggested impairment of synaptogenesis, viral toxicity in NSC/NPCs, and neural cell death to explain the neurological symptoms. However, it is not clear whether neurons, astrocytes, or microglia are infected by the virus or not. There are reports of sparse to no infection in microglia and cortical neurons, yet more investigation is required (Jacob et al., 2020; Kase and Okano, 2020; Zhang B. Z. et al., 2020; Ramani et al., 2021).

In addition to the role of organoids in exploring damages of tissue and somatic cells, they are accepted as valuable *ex vivo* models for studying stem cells. Accordingly, reports have revealed that the stem cells can be affected by SARS-CoV-2 as it decreases the population of resident stem cells in the lung. Some studies have reported that one type of pulmonary stem cell expresses ACE2, which can lead to its loss. Besides, intestinal organoid-based studies showed that both quiescent and active intestinal stem cells express ACE2. Therefore, investigating the exact role of the signaling pathways and susceptibility of stem cells to SARS-CoV-2 are essential in terms of finding potential treatments (Chugh et al., 2021).

The application of organoids in studying new variants of SAR-CoV-2 is another novel advantage of this model. The new variants including Delta and Lambda are more transmissible and can escape from the immune system and to some extent, the current vaccines. Organoid systems can provide a rapid platform for comparison of these strains and their susceptibility to vaccines and drugs (Mohammadi et al., 2021; van der Vaart et al., 2021). Modifying the organoids by using novel technologies like clustered regularly interspaced short palindromic repeats and CRISPR-associated protein9 (CRISPR/Cas9) and biomaterials as well as combining different cell types can result in more applicable and reliable pre-clinical models (Yu, 2021).

Furthermore, different animal models have helped researchers investigate body response and treatment options. Mice, hamsters, ferrets, and primates are the most common animal models and their characteristics were addressed previously. The size, rapid growth, and breeding of mice have made them favorable animal models in preclinical studies. On the other hand, the problem of their resistance to SARS-CoV-2 infection has raised some issues. The application of hACE2 transgene-dependent expression and adeno-associated virus delivery methods has helped scientists generate promising useful models. Nonetheless, there are still concerns about the similarity of hACE2 expression and distribution to humans and its related possible differences in modeling the SARS-CoV-2 infection mechanism. Hence, more specified manipulation of the relevant locus in mice may develop more accurate models. In contrast to mice, hamsters are known to be permissive to SARS-CoV-2. This model can be used for the mild form of COVID-19 and exploring the successful inflammation resolving mechanisms for therapeutic targets (Cleary et al., 2020; Kumar et al., 2020).

This review has discussed currently available models and their challenges in the paradigm of RM and proposed potential therapeutic approaches discovered in the experimental studies. Despite previous advances in this field, there are still shortcomings highlighted under the “CHALLENGES AND LIMITATIONS” section, which should be defeated in order to optimize COVID-19 RM modeling. For example, further exploration of gene editing or “omics” to improve the limitations of iPSC-based modeling such as immaturity and epigenetic memory retaining problems is suggested. However, economic efficiency should be considered in applying these new technologies. Altogether, considering the impossibility of studying the detailed mechanism of pathogenicity and the sequence of suggested drugs or vaccine candidates in human beings, these big steps toward RM in the SARS-CoV-2 field of study should be continued.

## Author Contributions

BL participated in the study design and drafting the manuscript. NF-H, MA, and AT-B contributed to the drafting of the manuscript. MR-T and HA provided final approval of the version to publish. BA supervised the project from the scientific view of point and advised on study design. All authors read, provided feedback, and approved the final manuscript.

## Conflict of Interest

The authors declare that the research was conducted in the absence of any commercial or financial relationships that could be construed as a potential conflict of interest.

## Publisher’s Note

All claims expressed in this article are solely those of the authors and do not necessarily represent those of their affiliated organizations, or those of the publisher, the editors and the reviewers. Any product that may be evaluated in this article, or claim that may be made by its manufacturer, is not guaranteed or endorsed by the publisher.

## Abbreviations

SARS-CoV-2: Severe Acute Respiratory Syndrome Coronavirus-2
MERS: Middle East Respiratory Syndrome
RM: Regenerative Medicine
hESCs: Human Embryonic Stem Cells
hiPSCs: Human Induced Pluripotent Stem Cells
MSCs: Mesenchymal Stem Cells
NSCs: Neural stem cells
NPCs: Neural progenitor cells
NK: Natural killer cells
AT2s: Type2 Alveolar Epithelial Cells
ARB: Angiotensin Receptor Blocker
hrsACE2: Human Recombinant Soluble ACE2
ARDS: Acute Respiratory Distress Syndrome
HSV1: Herpes Simplex virus-1
IFN: Interferon
HLA: Human Leukocyte Adhesion
hs-cTnI: Highly sensitive troponin-I
Cyr61: Cysteine-rich protein 61
CCN1: CCN family number 1
GSIs: γ-Secretase Inhibitors
GFP: Green Fluorescent Protein
SFTPC: Surfactant Protein-C
QNHC: Quinacrine Dihydrochloride
MPA: Mycophenolic Acid
CLDN1: claudin1
SLC10A2: solute carrier family 10 member 2
CFTR: Cystic Fibrosis Transmembrane Conductance Regulator
RLU,: Relative Luciferase Units
ECM: Extracellular Matrix
SCID: Severe Combined Immune Deficiency
NOD: Non-obese Diabetic
NSG: NOD-SCID iL2RG^*null*^
3D: 3 dimensional
hACE2: Human Angiotensin-Converting Enzyme 2
hPSC: Human Pluripotent Stem Cell
PDGFb: Platelet Derived Growth Factor Subunit B
KRT18: Cytokeratin 18
TMPRSS2: Transmembrane Serine Protease 2
RIG-I: Retinoic acid-Inducible Gene-I-like
PAMPs: Pathogen-Associated Molecular Patterns
Ang-1: Angiopoietin-1
KGF: Keratinocyte Growth Factor
BNP: Brain Natriuretic Peptide
MAS: Macrophage Activation Syndrome
ORF: Open Reading Frame
GM-CSF: Granulocyte-Macrophage Colony-Stimulating Factor
CXCL: C-X -C motif Chemokine Ligand
CXCL10/IP-10: C–X–C motif chemokine Ligand 10/Interferon γ-induced Protein 10 kDa
S protein: Spike
BMI: Body Mass Index
PD: Programmed cell Death protein
CD: Cluster of Differentiation
NSP: Non-structural Protein
M protein: Membrane
E protein: Envelope
N protein: Nucleocapsid
HCQ: Hydroxychloroquine
LPS: Lipopolysaccharide
DEX: Dexamethasone
TNF-a: Tumor Necrosis Factor Alpha
IL: Interleukin
NC: No Change
ARCoV: A vaccine candidate with the component of a mRNA sequence encoding the receptor binding domain of the virus which is encapsulated in lipid nanoparticles
RANTES: CCL5
Exo MSC-NTF: MCS-derived exosomes producing neurotrophic factor
AKI: Acute Kidney Injury
NIH: National Institutes of Health
WHO: World Health Organization
FDA: Food and Drug Administration
RAS: Renin–Angiotensin System
SIRS: Severe Inflammatory Response Syndrome
BBB: Blood- Brain Barrier
TAT: Thrombin Antithrombin complex
ABSCs: Airway Basal Stem Cells
CS: Cigarette Smoke
APC: Antigen Presenting Cells
STAT-1: Signal Transducer And Activator Of Transcription 1
JAK: Janus kinase
CRISPR/Cas9: Clustered Regularly Interspaced Short Palindromic Repeats and CRISPR-associated protein9
MASCp6: Mouse-Adapted Strain At Passage 6
Ifnar1-/- mice: C57BL/6 mice with a genetic ablation of their type I interferon receptors
Il28r-/- mice: C57BL/6 mice with a genetic ablation of their type III interferon receptors
WT: Wild Type
STAT2-/- hamster: lacking type I and III IFN signaling
IL28R hamster: lacking IFN type III signaling
ISG: Interferon-Stimulated Genes
GI: Gastrointestinal
PMN: Polymorphonuclear.

## References

[B1] Abo K. M. Ma L. Matte T. Huang J. Alysandratos K. D. Werder R. B. (2020). Human iPSC-derived alveolar and airway epithelial cells can be cultured at air-liquid interface and express SARS-CoV-2 host factors. *bioRxiv.* [Preprint].

[B2] Ahmad Mulyadi Lai H. I. Chou S.-J. Chien Y. Tsai P.-H. Chien C.-S. Hsu C.-C. (2021). Expression of Endogenous Angiotensin-Converting Enzyme 2 in Human Induced Pluripotent Stem Cell-Derived Retinal Organoids. *Int. J. Mol. Sci.* 22:1320. 10.3390/ijms22031320 33525682 PMC7865454

[B3] Akhmerov A. Marbán E. (2020). COVID-19 and the Heart. *Circ. Res.* 126 1443–1455. 32252591 10.1161/CIRCRESAHA.120.317055

[B4] Alsaad K. O. Arabi Y. M. Hajeer A. H. (2020). Spectrum of histopathological findings in coronavirus disease-19, Middle East respiratory syndrome and severe acute respiratory syndrome. *Ann. Thoracic Med.* 15 52–53. 10.4103/atm.atm_105_20 PMC7259397 32489438

[B5] Anderson R. H. Francis K. R. (2018). Modeling rare diseases with induced pluripotent stem cell technology. *Mol. Cell Probes.* 40 52–59. 10.1016/j.mcp.2018.01.001 29307697 PMC6033695

[B6] Arikan H. Ozturk S. Tokgoz B. Dursun B. Seyahi N. Trabulus S. (2021). Characteristics and outcomes of acute kidney injury in hospitalized COVID-19 patients: A multicenter study by the Turkish society of nephrology. *PLoS One* 16:e0256023. 10.1371/journal.pone.0256023 34375366 PMC8354466

[B7] Asgharzade S. Alizadeh A. Arab S. (2021). Regenerative Medicine Approaches in COVID-19 Pneumonia. *Curr. Stem Cell Res. Therapy* 16 647–655. 10.2174/1574888x16999210112205826 33438550

[B8] Atala A. Henn A. Lundberg M. Ahsan T. Greenberg J. Krukin J. (2020). Regen med therapeutic opportunities for fighting COVID−19. *Stem Cells Translat. Med.* 10 5–13. 10.1002/sctm.20-0245 32856432 PMC7461298

[B9] Basiri A. Pazhouhnia Z. Beheshtizadeh N. Hoseinpour M. Saghazadeh A. Rezaei N. (2020). Regenerative Medicine in COVID-19 Treatment: Real Opportunities and Range of Promises. *Stem Cell Rev. Rep.* 17 163–175. 10.1007/s12015-020-09994-5 32564256 PMC7305935

[B10] Becker R. C. (2020). COVID-19 update: Covid-19-associated coagulopathy. *J. Thromb. Thrombol.* 50 54–67. 10.1007/s11239-020-02134-3 32415579 PMC7225095

[B11] Belsky J. A. Tullius B. P. Lamb M. G. Sayegh R. Stanek J. R. Auletta J. J. (2021). COVID-19 in immunocompromised patients: A systematic review of cancer, hematopoietic cell and solid organ transplant patients. *J. Infect.* 82 329–338. 10.1016/j.jinf.2021.01.022 33549624 PMC7859698

[B12] Boudewijns R. Thibaut H. J. Kaptein S. J. Li R. Vergote V. Seldeslachts L. (2020). STAT2 signaling as double-edged sword restricting viral dissemination but driving severe pneumonia in SARS-CoV-2 infected hamsters. *BioRxiv.* [Preprint]. 10.1038/s41467-020-19684-y PMC7672082 33203860

[B13] Busnadiego I. Fernbach S. Pohl M. O. Karakus U. Huber M. Trkola A. (2020). Antiviral activity of type i, ii, and iii interferons counterbalances ace2 inducibility and restricts sars-cov-2. *MBio* 11 e1928–e1920. 10.1128/mBio.01928-20 PMC7484541 32913009

[B14] Cao X. (2020). COVID-19: immunopathology and its implications for therapy. *Nat. Rev. Immunol.* 20 269–270. 10.1038/s41577-020-0308-3 32273594 PMC7143200

[B15] Chigr F. Merzouki M. Najimi M. (2020). Autonomic brain centers and pathophysiology of COVID-19. *ACS Chem. Neurosci.* 11 1520–1522. 10.1021/acschemneuro.0c00265 32427468

[B16] Choi S. W. Shin J. S. Park S. J. Jung E. Park Y. G. Lee J. (2020). Antiviral activity and safety of remdesivir against SARS-CoV-2 infection in human pluripotent stem cell-derived cardiomyocytes. *Antiviral Res.* 184:104955. 10.1016/j.antiviral.2020.104955 33091434 PMC7571425

[B17] Chow Y.-H. O’Brodovich H. Plumb J. Wen Y. Sohn K.-J. Lu Z. (1997). Development of an epithelium-specific expression cassette with human DNA regulatory elements for transgene expression in lung airways. *PNAS* 94 14695–14700. 10.1073/pnas.94.26.14695 9405675 PMC25096

[B18] Chugh R. M. Bhanja P. Norris A. Saha S. (2021). Experimental Models to Study COVID-19 Effect in Stem Cells. *Cells* 10:91. 10.3390/cells10010091 33430424 PMC7827246

[B19] Cleary S. J. Pitchford S. C. Amison R. T. Carrington R. Robaina Cabrera C. L. Magnen M. (2020). Animal models of mechanisms of SARS-CoV-2 infection and COVID-19 pathology. *Br. J. Pharmacol.* 177 4851–4865. 10.1111/bph.15143 32462701 PMC7283621

[B20] Costela-Ruiz V. J. Illescas-Montes R. Puerta-Puerta J. M. Ruiz C. Melguizo-Rodríguez L. (2020). SARS-CoV-2 infection: The role of cytokines in COVID-19 disease. *Cytokine Growth Factor Rev.* 54 62–75. 32513566 10.1016/j.cytogfr.2020.06.001 PMC7265853

[B21] Covid-19 Treatment Guidelines Panel (2021). *Coronavirus Disease 2019 (COVID-19) Treatment Guidelines.* Bethesda, MD: National Institutes ofHealth. 34003615

[B22] Croy B. A. Di Santo J. P. Manz M. Bankert R. B. (2007). “Chapter 13 - Mouse Models of Immunodeficiency,” in *The Mouse in Biomedical Research*, 2nd Edn, eds Fox J. G. Davisson M. T. Quimby F. W. Barthold S. W. Newcomer C. E. Smith A. L. (Burlington: Academic Press), 275–289. 10.1016/b978-012369454-6/50091-1

[B23] Doss M. X. Sachinidis A. (2019). Current Challenges of iPSC-Based Disease Modeling and Therapeutic Implications. *Cells* 8:403. 10.3390/cells8050403 31052294 PMC6562607

[B24] Duan F. Guo L. Yang L. Han Y. Thakur A. Nilsson-Payant B. E. (2020). Modeling COVID-19 with Human Pluripotent Stem Cell-Derived Cells Reveals Synergistic Effects of Anti-inflammatory Macrophages with ACE2 Inhibition Against SARS-CoV-2. *Res. Square* 2020 3.rs–62758.rs.

[B25] Fehr A. R. Perlman S. (2015). Coronaviruses: an overview of their replication and pathogenesis. *Coronaviruses* 2015 1–23. 10.1007/978-1-0716-0900-2_1 PMC4369385 25720466

[B26] Ferreras C. Pascual-Miguel B. Mestre-Durán C. Navarro-Zapata A. Clares-Villa L. Martín-Cortázar C. (2021). SARS-CoV-2-Specific Memory T Lymphocytes From COVID-19 Convalescent Donors: Identification, Biobanking, and Large-Scale Production for Adoptive Cell Therapy. *Front. Cell Dev. Biol.* 9:620730. 10.3389/fcell.2021.620730 33718360 PMC7947351

[B27] Gibson P. G. Qin L. Puah S. H. (2020). COVID-19 acute respiratory distress syndrome (ARDS): clinical features and differences from typical pre-COVID-19 ARDS. *Med. J. Aust.* 213 54.e–56.e. 32572965 10.5694/mja2.50674 PMC7361309

[B28] Golchin A. Seyedjafari E. Ardeshirylajimi A. (2020). Mesenchymal Stem Cell Therapy for COVID-19: Present or Future. *Stem Cell Rev. Rep.* 2020 1–7. 10.1007/s12015-020-09973-w PMC7152513 32281052

[B29] Han Y. Duan X. Yang L. Nilsson-Payant B. E. Wang P. Duan F. (2021). Identification of SARS-CoV-2 inhibitors using lung and colonic organoids. *Nature* 589 270–275. 33116299 10.1038/s41586-020-2901-9 PMC8034380

[B30] Harmer D. Gilbert M. Borman R. Clark K. L. (2002). Quantitative mRNA expression profiling of ACE 2, a novel homologue of angiotensin converting enzyme. *FEBS Lett.* 532 107–110. 10.1016/s0014-5793(02)03640-2 12459472

[B31] Harrison A. G. Lin T. Wang P. (2020). Mechanisms of SARS-CoV-2 Transmission and Pathogenesis. *Trends Immunol.* 41 1100–1115. 10.1016/j.it.2020.10.004 33132005 PMC7556779

[B32] Huang J. Hume A. J. Abo K. M. Werder R. B. Villacorta-Martin C. Alysandratos K.-D. (2020). SARS-CoV-2 Infection of Pluripotent Stem Cell-Derived Human Lung Alveolar Type 2 Cells Elicits a Rapid Epithelial-Intrinsic Inflammatory Response. *Cell Stem Cell* 27 962.e–973.e. 32979316 10.1016/j.stem.2020.09.013 PMC7500949

[B33] Hue S. Beldi-Ferchiou A. Bendib I. Surenaud M. Fourati S. Frapard T. (2020). Uncontrolled Innate and Impaired Adaptive Immune Responses in Patients with COVID-19 Acute Respiratory Distress Syndrome. *Am. J. Respirat. Crit. Care Med.* 202 1509–1519. 10.1164/rccm.202005-1885oc 32866033 PMC7706149

[B34] Jacob F. Pather S. R. Huang W. K. Zhang F. Wong S. Z. H. Zhou H. (2020). Human Pluripotent Stem Cell-Derived Neural Cells and Brain Organoids Reveal SARS-CoV-2 Neurotropism Predominates in Choroid Plexus Epithelium. *Cell Stem Cell* 27 937.e–950.e. 33010822 10.1016/j.stem.2020.09.016 PMC7505550

[B35] Kase Y. Okano H. (2020). Expression of ACE2 and a viral virulence-regulating factor CCN family member 1 in human iPSC-derived neural cells: implications for COVID-19-related CNS disorders. *Inflammat. Regenerat.* 40:32. 10.1186/s41232-020-00143-6 PMC7485212 32934757

[B36] Kaspi H. Semo J. Abramov N. Dekel C. Lindborg S. Kern R. (2021). MSC-NTF (NurOwn^®^) exosomes: a novel therapeutic modality in the mouse LPS-induced ARDS model. *Stem Cell Res. Therapy* 12 1–10. 10.1155/2019/6458237 33468250 PMC7814377

[B37] Kasuga Y. Zhu B. Jang K. J. Yoo J. S. (2021). Innate immune sensing of coronavirus and viral evasion strategies. *Exp. Mol. Med.* 53 723–736. 10.1038/s12276-021-00602-1 33953325 PMC8099713

[B38] Katsura H. Sontake V. Tata A. Kobayashi Y. Edwards C. E. Heaton B. E. (2020). Human Lung Stem Cell-Based Alveolospheres Provide Insights into SARS-CoV-2-Mediated Interferon Responses and Pneumocyte Dysfunction. *Cell Stem Cell* 27 890.e–904.e. 33128895 10.1016/j.stem.2020.10.005 PMC7577733

[B39] Khoury M. Cuenca J. Cruz F. F. Figueroa F. E. Rocco P. R. M. Weiss D. J. (2020). Current status of cell-based therapies for respiratory virus infections: applicability to COVID-19. *Eur. Respir. J.* 55:2000858. 10.1183/13993003.00858-2020 32265310 PMC7144273

[B40] Krüger J. Groß R. Conzelmann C. Müller J. A. Koepke L. Sparrer K. M. J. (2021). Drug Inhibition of SARS-CoV-2 Replication in Human Pluripotent Stem Cell–Derived Intestinal Organoids. *Cell. Mol. Gastroenterol. Hepatol.* 11 935–948. 10.1016/j.jcmgh.2020.11.003 33186749 PMC7655023

[B41] Kumar S. Yadav P. K. Srinivasan R. Perumal N. (2020). Selection of animal models for COVID-19 research. *VirusDisease* 31 453–458. 10.1007/s13337-020-00637-4 33283030 PMC7709475

[B42] Lamers M. M. Beumer J. van der Vaart J. Knoops K. Puschhof J. Breugem T. I. (2020). SARS-CoV-2 productively infects human gut enterocytes. *Science* 369 50–54. 10.1126/science.abc1669 32358202 PMC7199907

[B43] Lamers M. M. van der Vaart J. Knoops K. Riesebosch S. Breugem T. I. Mykytyn A. Z. (2021). An organoid-derived bronchioalveolar model for SARS-CoV-2 infection of human alveolar type II-like cells. *EMBO J.* 40:e105912. 10.15252/embj.2020105912 PMC7883112 33283287

[B44] Legrand M. Bell S. Forni L. Joannidis M. Koyner J. L. Liu K. (2021). Pathophysiology of COVID-19-associated acute kidney injury. *Nat. Rev. Nephrol.* 2021 1–14. 10.1038/s41581-021-00452-0 PMC8256398 34226718

[B45] Lei X. Dong X. Ma R. Wang W. Xiao X. Tian Z. (2020). Activation and evasion of type I interferon responses by SARS-CoV-2. *Nat. Commun.* 11:3810. 10.1038/s41467-020-17665-9 PMC7392898 32733001

[B46] Li J. Fan J.-G. (2020). Characteristics and Mechanism of Liver Injury in 2019 Coronavirus Disease. *J. Clin. Translat. Hepatol.* 8 13–17. 10.14218/JCTH.2020.00019 PMC7132021 32274341

[B47] Li X. Geng M. Peng Y. Meng L. Lu S. (2020). Molecular immune pathogenesis and diagnosis of COVID-19. *J. Pharmaceut. Anal.* 10 102–108. 10.1016/j.jpha.2020.03.001 32282863 PMC7104082

[B48] Lui V. Hui K. Ottakandathil Babu R. Yue H. Chung P. H. Y. Tam P. (2021). Human liver organoid derived intra-hepatic bile duct cells support SARS-CoV-2 infection and replication and its comparison with SARS-CoV2021. *SARS.* [Preprint]:1251458 10.1038/s41598-022-09306-6 PMC8965546 35354880

[B49] Mahalingam R. Dharmalingam P. Santhanam A. Kotla S. Davuluri G. Karmouty-Quintana H. (2021). Single-cell RNA sequencing analysis of SARS-CoV-2 entry receptors in human organoids. *J. Cell. Physiol.* 236 2950–2958. 10.1002/jcp.30054 32944935 PMC7537521

[B50] Makovoz B. Moeller R. Zebitz Eriksen A. tenOever B. R. Blenkinsop T. A. (2020). SARS-CoV-2 Infection of Ocular Cells from Human Adult Donor Eyes and hESC-Derived Eye Organoids. *SSRN* 2020:3650574.

[B51] Mallapaty S. (2021). The mini lungs and other organoids helping to beat COVID. *Nature* 593 492–494. 10.1038/d41586-021-01395-z 34040221

[B52] Mohammadi M. Shayestehpour M. Mirzaei H. (2021). The impact of spike mutated variants of SARS-CoV2 [Alpha, Beta, Gamma, Delta, and Lambda] on the efficacy of subunit recombinant vaccines. *Brazil. J. Infect. Dis.* 2021:101606. 10.1016/j.bjid.2021.101606 34428473 PMC8367756

[B53] Molinaro R. Pasto A. Taraballi F. Giordano F. Azzi J. A. Tasciotti E. (2020). Biomimetic Nanoparticles Potentiate the Anti-Inflammatory Properties of Dexamethasone and Reduce the Cytokine Storm Syndrome: An Additional Weapon against COVID-19? *Nanomaterials* 10:2301. 10.3390/nano10112301 33233748 PMC7699958

[B54] Monteil V. Kwon H. Prado P. Hagelkrüys A. Wimmer R. A. Stahl M. (2020). Inhibition of SARS-CoV-2 Infections in Engineered Human Tissues Using Clinical-Grade Soluble Human ACE2. *Cell* 181 905.e–913.e. 32333836 10.1016/j.cell.2020.04.004 PMC7181998

[B55] Moriguchi T. Harii N. Goto J. Harada D. Sugawara H. Takamino J. (2020). A first case of meningitis/encephalitis associated with SARS-Coronavirus-2. *Int. J. Infect. Dis.* 94 55–58. 32251791 10.1016/j.ijid.2020.03.062 PMC7195378

[B56] Ng O.-W. Chia A. Tan A. T. Jadi R. S. Leong H. N. Bertoletti A. (2016). Memory T cell responses targeting the SARS coronavirus persist up to 11 years post-infection. *Vaccine* 34 2008–2014. 10.1016/j.vaccine.2016.02.063 26954467 PMC7115611

[B57] No Author. (2020). Biggest COVID-19 trial tests repurposed drugs first. *Nat. Biotechnol.* 38:510. 10.1038/s41587-020-0528-x 32393915

[B58] Nolasco P. Borsoi J. Moraes C. B. Freitas-Junior L. H. Pereira L. V. (2020). Human induced pluripotent stem cells as a tool for disease modeling and drug screening for COVID-19. *Genet. Mol. Biol.* 44 (1 Suppl. 1):e20200198. 10.1590/1678-4685-GMB-2020-0198 PMC7737100 33275129

[B59] Otsuka R. Seino K.-I. (2020). Macrophage activation syndrome and COVID-19. *Inflammat. Regenerat.* 40 1–6. 10.1016/j.trsl.2021.03.002 32834892 PMC7406680

[B60] Park S. J. Yu K. M. Kim Y. I. Kim S. M. Kim E. H. Kim S. G. (2020). Antiviral Efficacies of FDA-Approved Drugs against SARS-CoV-2 Infection in Ferrets. *mBio* 11 e1114–e1120. 10.1128/mBio.01114-20 PMC7244896 32444382

[B61] Park Y. J. Farooq J. Cho J. Sadanandan N. Cozene B. Gonzales-Portillo B. (2021). Fighting the War Against COVID-19 via Cell-Based Regenerative Medicine: Lessons Learned from 1918 Spanish Flu and Other Previous Pandemics. *Stem Cell Rev. Rep.* 17 9–32. 10.1007/s12015-020-10026-5 32789802 PMC7423503

[B62] Payab M. Abedi M. Foroughi Heravani N. Hadavandkhani M. Arabi M. Tayanloo-Beik A. (2021). Brown adipose tissue transplantation as a novel alternative to obesity treatment: a systematic review. *Int. J. Obes.* 45 109–121. 10.1038/s41366-020-0616-5 32499525

[B63] Pellegrini L. Albecka A. Mallery D. L. Kellner M. J. Paul D. Carter A. P. (2020). SARS-CoV-2 Infects the Brain Choroid Plexus and Disrupts the Blood-CSF Barrier in Human Brain Organoids. *Cell Stem Cell* 27 951.e–961.e. 33113348 10.1016/j.stem.2020.10.001 PMC7553118

[B64] Pérez-Martínez A. Mora-Rillo M. Ferreras C. Guerra-García P. Pascual-Miguel B. Mestre-Durán C. (2021). Phase I dose-escalation single centre clinical trial to evaluate the safety of infusion of memory T cells as adoptive therapy in COVID-19 (RELEASE). *EClinicalMedicine* 39:101086. 10.1016/j.eclinm.2021.101086 34405140 PMC8361305

[B65] Petrovic V. Radenkovic D. Radenkovic G. Djordjevic V. Banach M. (2020). Pathophysiology of cardiovascular complications in COVID-19. *Front. Physiol.* 11:575600. 10.3389/fphys.2020.575600 33162899 PMC7583694

[B66] Pirraglia M. P. Ceccarelli G. Cerini A. Visioli G. d’Ettorre G. Mastroianni C. M. (2020). Retinal involvement and ocular findings in COVID-19 pneumonia patients. *Sci. Rep.* 10 1–7. 33060700 10.1038/s41598-020-74446-6 PMC7566835

[B67] Pourbagheri-Sigaroodi A. Bashash D. Olfatifar M. Salari S. Abolghasemi H. (2020). What We Know of the Prognostic Value of Lymphopenia in SARS-CoV-2 Infection. *IJBC* 12 75–79.

[B68] Poyiadji N. Shahin G. Noujaim D. Stone M. Patel S. Griffith B. (2020). COVID-19–associated Acute Hemorrhagic Necrotizing Encephalopathy: Imaging Features. *Radiology* 296 E119–E120. 32228363 10.1148/radiol.2020201187 PMC7233386

[B69] Prior N. Inacio P. Huch M. (2019). Liver organoids: from basic research to therapeutic applications. *Gut* 68 2228–2237. 10.1136/gutjnl-2019-319256 31300517 PMC6872443

[B70] Puelles V. G. Lütgehetmann M. Lindenmeyer M. T. Sperhake J. P. Wong M. N. Allweiss L. (2020). Multiorgan and renal tropism of SARS-CoV-2. *N. Engl. J. Med.* 383 590–592. 32402155 10.1056/NEJMc2011400 PMC7240771

[B71] Pujari R. Chan G. Tapply I. Bourne R. R. (2021). The impacts of COVID-19 on glaucoma patient outcomes as assessed by POEM. *Eye* 2021 1–3. 10.1038/s41433-021-01425-0 PMC7871516 33564139

[B72] Purkayastha A. Sen C. Garcia G. Langerman J. Shia D. W. Meneses L. K. (2020). Direct Exposure to SARS-CoV-2 and Cigarette Smoke Increases Infection Severity and Alters the Stem Cell-Derived Airway Repair Response. *Cell Stem Cell* 27 869.e–875.e. 33259798 10.1016/j.stem.2020.11.010 PMC7670932

[B73] Qin H. Zhao A. (2020). Mesenchymal stem cell therapy for acute respiratory distress syndrome: from basic to clinics. *Protein Cell* 11 707–722. 10.1007/s13238-020-00738-2 32519302 PMC7282699

[B74] Rahim R. A. Muhammad N. (2020). The Pursuit of Covid-19 Animal Models. *IIUM Medical J. Malaysia* 19:1610.

[B75] Ramani A. Müller L. Ostermann P. N. Gabriel E. Abida-Islam P. Müller-Schiffmann A. (2020). SARS-CoV-2 targets neurons of 3D human brain organoids. *EMBO J.* 39:e106230. 10.15252/embj.2020106230 PMC7560208 32876341

[B76] Ramani A. Pranty A. I. Gopalakrishnan J. (2021). Neurotropic Effects of SARS-CoV-2 Modeled by the Human Brain Organoids. *Stem Cell Rep.* 16 373–384. 10.1016/j.stemcr.2021.02.007 33631123 PMC7879157

[B77] Ramezankhani R. Solhi R. Memarnejadian A. Nami F. Hashemian S. M. R. Tricot T. (2020). Therapeutic modalities and novel approaches in regenerative medicine for COVID-19. *Int. J. Antimicrob. Agents* 56:106208. 10.1016/j.ijantimicag.2020.106208 33213829 PMC7582055

[B78] Remy K. E. Mazer M. Striker D. A. Ellebedy A. H. Walton A. H. Unsinger J. (2020). Severe immunosuppression and not a cytokine storm characterizes COVID-19 infections. *JCI Insight* 5:e140329. 10.1172/jci.insight.140329 PMC7526441 32687484

[B79] Robbins-Juarez S. Y. Qian L. King K. L. Stevens J. S. Husain S. A. Radhakrishnan J. (2020). Outcomes for patients with COVID-19 and acute kidney injury: a systematic review and meta-analysis. *Kidney Int. Rep.* 5 1149–1160. 10.1016/j.ekir.2020.06.013 32775814 PMC7314696

[B80] Rodriguez-Guerra M. Jadhav P. Vittorio T. J. (2021). Current treatment in COVID-19 disease: a rapid review. *Drugs Context* 10 2020–2010. 10.7573/dic.2020-10-3 PMC7850293 33569082

[B81] Salahudeen A. A. Choi S. S. Rustagi A. Zhu J. van Unen V. de la O. S. (2020). Progenitor identification and SARS-CoV-2 infection in human distal lung organoids. *Nature* 588 670–675. 33238290 10.1038/s41586-020-3014-1 PMC8003326

[B82] Sánchez-Guijo F. García-Arranz M. López-Parra M. Monedero P. Mata-Martínez C. Santos A. (2020). Adipose-derived mesenchymal stromal cells for the treatment of patients with severe SARS-CoV-2 pneumonia requiring mechanical ventilation. A proof of concept study. *EClinicalMedicine* 25:100454. 10.1016/j.eclinm.2020.100454 32838232 PMC7348610

[B83] Seah I. Agrawal R. (2020). Can the coronavirus disease 2019 (COVID-19) affect the eyes? A review of coronaviruses and ocular implications in humans and animals. *Ocular Immunol. Inflammat.* 28 391–395. 10.1080/09273948.2020.1738501 32175797 PMC7103678

[B84] Shang J. Wan Y. Luo C. Ye G. Geng Q. Auerbach A. (2020). Cell entry mechanisms of SARS-CoV-2. *Proc. Natl. Acad. Sci.* 117 11727–11734. 32376634 10.1073/pnas.2003138117 PMC7260975

[B85] Sharma A. Garcia G. Wang Y. Plummer J. T. Morizono K. Arumugaswami V. (2020). Human iPSC-Derived Cardiomyocytes Are Susceptible to SARS-CoV-2 Infection. *Cell Rep. Med.* 1:100052. 10.1016/j.xcrm.2020.100052 32835305 PMC7323681

[B86] Shi J. Wen Z. Zhong G. Yang H. Wang C. Huang B. (2020). Susceptibility of ferrets, cats, dogs, and other domesticated animals to SARS-coronavirus 2. *Science* 368 1016–1020. 10.1126/science.abb7015 32269068 PMC7164390

[B87] Shpichka A. Bikmulina P. Peshkova M. Kosheleva N. Zurina I. Zahmatkesh E. (2020). Engineering a Model to Study Viral Infections: Bioprinting, Microfluidics, and Organoids to Defeat Coronavirus Disease 2019 (COVID-19). *Int. J. Bioprint* 6:302. 10.18063/ijb.v6i4.302 PMC7557357 33089000

[B88] Simoneau C. R. Ott M. (2020). Modeling Multi-organ Infection by SARS-CoV-2 Using Stem Cell Technology. *Cell Stem Cell* 27 859–868. 10.1016/j.stem.2020.11.012 33275899 PMC7713543

[B89] Song E. Zhang C. Israelow B. Lu-Culligan A. Prado A. V. Skriabine S. (2021). Neuroinvasion of SARS-CoV-2 in human and mouse brain. *J. Exp. Med.* 218:e20202135 10.1084/jem.20202135 PMC7808299 33433624

[B90] Steardo L. Steardo L. Jr. Zorec R. Verkhratsky A. (2020). Neuroinfection may contribute to pathophysiology and clinical manifestations of COVID-19. *Acta Physiol.* 229 e13473–e. 10.1111/apha.13473 PMC7228251 32223077

[B91] Sterneckert J. Reinhardt P. Schöler H. (2014). Investigating human disease using stem cell models. *Nat. Rev. Genet.* 15 625–639. 10.1038/nrg3764 25069490

[B92] Sun J. Zhuang Z. Zheng J. Li K. Wong R. L. Liu D. (2020). Generation of a Broadly Useful Model for COVID-19 Pathogenesis, Vaccination, and Treatment. *Cell* 182 734.e–743.e. 32643603 10.1016/j.cell.2020.06.010 PMC7284240

[B93] Suri J. S. Agarwal S. Gupta S. K. Puvvula A. Biswas M. Saba L. (2021). A narrative review on characterization of acute respiratory distress syndrome in COVID-19-infected lungs using artificial intelligence. *Comput. Biol. Med.* 130:104210. 10.1016/j.compbiomed.2021.104210 PMC7813499 33550068

[B94] ter Meulen J. Bakker A. B. van den Brink E. N. Weverling G. J. Martina B. E. Haagmans B. L. (2004). Human monoclonal antibody as prophylaxis for SARS coronavirus infection in ferrets. *Lancet* 363 2139–2141. 10.1016/s0140-6736(04)16506-9 15220038 PMC7112500

[B95] Tindle C. Fuller M. Fonseca A. Taheri S. Ibeawuchi S.-R. Beutler N. (2021). Adult Stem Cell-derived Complete Lung Organoid Models Emulate Lung Disease in COVID-19. *Elife* 10:e66417. 10.7554/eLife.66417 PMC8463074 34463615

[B96] van der Vaart J. Lamers M. M. Haagmans B. L. Clevers H. (2021). Advancing lung organoids for COVID-19 research. *Dis. Models Mechanis.* 14:dmm049060. 10.1242/dmm.049060 PMC8272930 34219165

[B97] Wang L. Zhang Y. Zhang S. (2020). Cardiovascular impairment in COVID-19: learning from current options for cardiovascular anti-inflammatory therapy. *Front. Cardiovasc. Med.* 7:78. 10.3389/fcvm.2020.00078 32426374 PMC7203508

[B98] Wiersinga W. J. Rhodes A. Cheng A. C. Peacock S. J. Prescott H. C. (2020). Pathophysiology, Transmission, Diagnosis, and Treatment of Coronavirus Disease 2019 (COVID-19): A Review. *JAMA* 324 782–793. 10.1001/jama.2020.12839 32648899

[B99] Wong C. K. Luk H. K. Lai W. H. Lau Y. M. Zhang R. R. Wong A. C. (2020). Human-Induced Pluripotent Stem Cell-Derived Cardiomyocytes Platform to Study SARS-CoV-2 Related Myocardial Injury. *Circ. J.* 84 2027–2031. 10.1253/circj.cj-20-0881 32981925

[B100] Xia S. Wu M. Chen S. Zhang T. Ye L. Liu J. (2020). Long Term Culture of Human Kidney Proximal Tubule Epithelial Cells Maintains Lineage Functions and Serves as an Ex vivo Model for Coronavirus Associated Kidney Injury. *Virol. Sin.* 35 311–320. 10.1007/s12250-020-00253-y 32602046 PMC7322379

[B101] Yang L. Han Y. Nilsson-Payant B. E. Gupta V. Wang P. Duan X. (2020). A Human Pluripotent Stem Cell-based Platform to Study SARS-CoV-2 Tropism and Model Virus Infection in Human Cells and Organoids. *Cell Stem Cell* 27 125.e–136.e. 32579880 10.1016/j.stem.2020.06.015 PMC7303620

[B102] Yi S. A. Nam K. H. Yun J. Gim D. Joe D. Kim Y. H. (2020). Infection of Brain Organoids and 2D Cortical Neurons with SARS-CoV-2 Pseudovirus. *Viruses* 12:1004. 10.3390/v12091004 32911874 PMC7551632

[B103] Yiangou L. Davis R. P. Mummery C. L. (2020). Using Cardiovascular Cells from Human Pluripotent Stem Cells for COVID-19 Research: Why the Heart Fails. *Stem Cell Rep.* 16 385–397. 10.1016/j.stemcr.2020.11.003 33306986 PMC7833904

[B104] Youhanna S. Wright S. C. Lauschke V. M. (2021). Organotypic human ex vivo models for coronavirus disease 2019 research and drug development. *Curr. Opin. Pharmacol.* 59 11–18. 10.1016/j.coph.2021.04.006 34029832 PMC8075816

[B105] Youk J. Kim T. Evans K. V. Jeong Y.-I. Hur Y. Hong S. P. (2020). Three-Dimensional Human Alveolar Stem Cell Culture Models Reveal Infection Response to SARS-CoV-2. *Cell Stem Cell* 27 905.e–919.e. 33142113 10.1016/j.stem.2020.10.004 PMC7577700

[B106] Yu F. Jia R. Tang Y. Liu J. Wei B. (2020). SARS-CoV-2 infection and stem cells: interaction and intervention. *Stem Cell Res.* 2020:101859. 10.1016/j.scr.2020.101859 32570174 PMC7263221

[B107] Yu J. (2021). Organoids: A New Model for SARS-CoV-2 Translational Research. *Int. J. Stem Cells* 14 138–149. 10.15283/ijsc20169 33632991 PMC8138661

[B108] Zhang B.-Z. Chu H. Han S. Shuai H. Deng J. Hu Y.-F. (2020). SARS-CoV-2 infects human neural progenitor cells and brain organoids. *Cell Res.* 30 928–931. 10.1038/s41422-020-0390-x 32753756 PMC7399356

[B109] Zhang C. Shi L. Wang F.-S. (2020). Liver injury in COVID-19: management and challenges. *Lancet Gastroenterol. Hepatol.* 5 428–430. 10.1016/s2468-1253(20)30057-1 32145190 PMC7129165

[B110] Zhang J. Garrett S. Sun J. (2020). Gastrointestinal symptoms, pathophysiology, and treatment in COVID-19. *Genes Dis.* 8 385–400. 10.1016/j.gendis.2020.08.013 33521210 PMC7836435

[B111] Zhang N.-N. Li X.-F. Deng Y.-Q. Zhao H. Huang Y.-J. Yang G. (2020). A Thermostable mRNA Vaccine against COVID-19. *Cell* 182 1271.e–1283.e. 32795413 10.1016/j.cell.2020.07.024 PMC7377714

[B112] Zhao B. Ni C. Gao R. Wang Y. Yang L. Wei J. (2020). Recapitulation of SARS-CoV-2 infection and cholangiocyte damage with human liver ductal organoids. *Protein Cell* 11 771–775. 10.1007/s13238-020-00718-6 32303993 PMC7164704

[B113] Zheng J. Wong L.-Y. R. Li K. Verma A. K. Ortiz M. E. Wohlford-Lenane C. (2021). COVID-19 treatments and pathogenesis including anosmia in K18-hACE2 mice. *Nature* 589 603–607. 10.1038/s41586-020-2943-z 33166988 PMC7855185

